# Complex Transcriptional Profiles of the *PPP1R12A* Gene in Cells of the Circulatory System as Revealed by In Silico Analysis and Reverse Transcription PCR

**DOI:** 10.3390/cells11152315

**Published:** 2022-07-27

**Authors:** Paulo André Saldanha, Israel Olapeju Bolanle, Timothy Martin Palmer, Leonid Leonidovich Nikitenko, Francisco Rivero

**Affiliations:** Centre for Biomedicine, Hull York Medical School, Faculty of Health Sciences, University of Hull, Hull HU6 7RX, UK; paulo.saldanha@hyms.ac.uk (P.A.S.); olapeju.bolanle@hyms.ac.uk (I.O.B.); tim.palmer@hyms.ac.uk (T.M.P.); l.nikitenko@hull.ac.uk (L.L.N.)

**Keywords:** MYPT1, *PPP1R12A*, alternative splicing, promoter, terminator, HUVEC, HSVSMC, platelet

## Abstract

The myosin light chain phosphatase target subunit 1 (MYPT1), encoded by the *PPP1R12A* gene, is a key component of the myosin light chain phosphatase (MLCP) protein complex. MYPT1 isoforms have been described as products of the cassette-type alternative splicing of exons E13, E14, E22, and E24. Through in silico analysis of the publicly available EST and mRNA databases, we established that *PPP1R12A* contains 32 exons (6 more than the 26 previously reported), of which 29 are used in 11 protein-coding transcripts. An in silico analysis of publicly available RNAseq data combined with validation by reverse transcription (RT)-PCR allowed us to determine the relative abundance of each transcript in three cell types of the circulatory system where MYPT1 plays important roles: human umbilical vein endothelial cells (HUVEC), human saphenous vein smooth muscle cells (HSVSMC), and platelets. All three cell types express up to 10 transcripts at variable frequencies. HUVECs and HSVSMCs predominantly express the full-length variant (58.3% and 64.3%, respectively) followed by the variant skipping E13 (33.7% and 23.1%, respectively), whereas in platelets the predominant variants are those skipping E14 (51.4%) and E13 (19.9%), followed by the full-length variant (14.4%). Variants including E24 account for 5.4% of transcripts in platelets but are rare (<1%) in HUVECs and HSVSMCs. Complex transcriptional profiles were also found across organs using in silico analysis of RNAseq data from the GTEx project. Our findings provide a platform for future studies investigating the specific (patho)physiological roles of understudied MYPT1 isoforms.

## 1. Introduction

The 110–133 kDa protein phosphatase 1 regulatory subunit 12A, commonly known as myosin light chain phosphatase target subunit 1 (MYPT1), is a key regulator of the protein phosphatase 1c (PP1c), specifically the β/δ isoform. Together with a small subunit of 20–21 kDa (M20/21) of unclear function, they comprise the myosin light chain phosphatase (MLCP) holoenzyme. In this complex, MYPT1 acts as a scaffold that brings PP1c into close proximity with its substrates [1,2]. MYPT1 contains several structural motifs, including a myosin phosphatase N-terminal element (MyPhoNE; residues 10–17), a K35VKF38 motif and several ankyrin repeats at the N-terminus that facilitate the interaction with PP1c, and a leucine zipper (LZ) domain at the C-terminus that is required for interaction with protein kinase G (PKG) and subsequent activation of MLCP by cGMP signaling [3].

The activity, protein-protein interactions, and localization of MLCP are fine-tuned by the phosphorylation status of MYPT1, which is targeted by multiple protein kinases [3]. MLCP has an established role in regulating actomyosin contractility by antagonizing the effect of the myosin light chain kinase (MLCK). This role has been studied extensively in smooth muscle cells (blood vessels, uterus, chicken gizzard), where MYPT1 is a key target of signaling pathways that control smooth muscle tone [4]. In non-muscle cells, MLCP contributes to the regulation of cell shape, cell migration, and cell adhesion, as well as epithelial and endothelial barrier function [5,6,7,8]. In addition, MYPT1 participates in the regulation of critical cellular functions, such as cell proliferation, mitosis, synaptic vesicle release, and gene expression (reviewed in [1]).

The gene encoding MYPT1 (*PPP1R12A* in humans) is conserved in metazoans, and in vertebrates it consists of at least 26 protein-coding exons [9]. MYPT1 isoforms have been described as the product of the cassette-type alternative splicing of exons at two regions, the central part (referred to as the central insert, CI) and the 3′-end of the transcript (involving exon 24, E24) [10,11,12,13,14,15,16,17,18,19]. Skipping of E24 results in an isoform with a C-terminal leucine zipper (LZ) motif, whereas the inclusion of the exon causes a frame shift and introduces a premature stop codon, resulting in a shorter isoform that lacks the LZ motif and is less sensitive to nitric oxide signaling [9]. The LZ^−^ splice variant is subject to developmental regulation and is predominantly expressed in phasic (rhythmically contracting) smooth muscle cells such as those in the bladder, but it is less abundant in the tonic (continuously contracted) smooth muscle cells of the large arteries and is absent in most other tissues [10,11,12]. E24 variants have been extensively studied in chickens and rodents for their role in smooth muscle physiology [2,13,14]. In humans, the LZ^−^ variant, although not annotated in GenBank, has been reported in the smooth muscle of several vascular beds, bladder, and uterus [12,15,16].

CI splice variants were initially reported in chickens (involving E12), where MYPT1 was first characterized [17], and subsequently in rats (involving E13 and E14) and human cells (involving E13) [5,18]. Five rat CI variants arising by cassette-type alternative splicing and the usage of alternative splicing acceptor sites have been described [10,19]. CI variants have not been extensively studied and their functional relevance is unclear, but a lower rate of PKG-mediated phosphorylation in vitro has been reported in the chicken isoform skipping E12 [20], and a human isoform skipping E13 was proposed to have a lower binding affinity for radixin [7] compared to the full-length isoform. No other *PPP1R12A* variants have been reported other than a splice variant of E22 in a GenBank submission, which was found to be absent in HeLa and vascular endothelial cells using a PCR approach [7].

The existence of alternative splicing variants of *PPP1R12A* and orthologous genes is well documented. In addition, Machida et al. [21] analyzed the 5′ region of *PPP1R12A* in aortic smooth muscle and experimentally determined a transcription initiation site. However, an inspection of the information available in public databases indicates the presently unappreciated transcriptional complexity of this gene. While GenBank reports just 5 *PPP1R12A* transcripts encoding four protein isoforms, Ensembl (accession number ENSG00000058272) collates the sequence information from EST and mRNA submissions into 23 transcripts categorized as protein-coding, nonsense-mediated decay, and retained intron. In addition, much of the published information on *PPP1R12A* expression and transcription variants has been gained from studies in a limited number of organs and tissues, which consist of a mixture of cell types. Studies on cell lines or individual cell types are scarce and of limited scope [5,7,18].

To begin filling these knowledge gaps, we have undertaken a comprehensive examination of the transcriptional landscape of the *PPP1R12A* gene, with a focus on protein-coding transcripts. Through in silico analyses of GenBank and Ensembl data, we have generated a comprehensive profile of transcripts and determined the promoter and terminator regions of *PPP1R12A.* We have analyzed publicly available RNAseq data to determine the relative abundance of each transcript in three cell types of the circulatory system in which MYPT1 is known to play important roles, namely endothelial cells (represented by HUVECs) [7,22,23,24,25], smooth muscle cells (represented by HSVSMCs) [4], and platelets [26,27], and have verified our findings with a reverse transcription (RT)-PCR approach. In addition, we have determined *PPP1R12A* transcriptional profiles across organs using in silico analyses of RNAseq data from the GTEx project [28]. Our analyses revealed a previously unrecognized complex transcriptional repertoire for the human *PPP1R12A* gene that we expect will inform future studies examining the roles of specific MYPT1 variants in heath and disease.

## 2. Materials and Methods

### 2.1. Reagents

Unless otherwise indicated, reagents were obtained from Merck (Poole, UK) and Melford Laboratories (Ipswich, UK).

### 2.2. In Silico Analyses

Data for in silico analyses of *PPP1R12A* transcripts were obtained from GenBank^®^ (https://www.ncbi.nlm.nih.gov/genbank/), Ensembl (https://www.ensembl.org/index.html), the Genotype-Tissue Expression (GTEx) project portal (https://www.gtexportal.org/home/), and the AceView portal (https://www.ncbi.nlm.nih.gov/IEB/Research/Acembly/index.html) (all last accessed on 22 May 2022). AceView collates all public mRNA sequences: mRNAs from GenBank or RefSeq, single pass cDNA sequences from dbEST and Trace, and deep transcriptomics sequences from the Sequence Read Archive (SRA) and Gene Expression Omnibus (GEO) [29]. It was last updated in October 2012. GTEx is a resource to study tissue-specific gene expression and regulation from samples collected from 54 non-diseased tissue sites across nearly 1000 individuals [28]. GTEx is the only resource that provides data on individual exons and intron–exon boundaries for each tissue. Data from the release V8 of August 2019 were used. RNAseq data from GenBank were collected from the RNAseq intron features window of the *PPP1R12A* gene page (update 8 April 2022). As the data from AceView, GTEx, and GenBank do not overlap, they were combined to calculate the frequency of alternative splicing of exons of interest. PROMO (http://alggen.lsi.upc.es/cgi-bin/promo_v3/promo/promoinit.cgi?dirDB=TF_8.3; last accessed 12 January 2022) was used for transcription factor binding site identification using version 8.3 of TRANSFAC [30]. The non-redundant EST and SRA databases were interrogated with Blastn searches (https://blast.ncbi.nlm.nih.gov/Blast.cgi; last accessed 11th May 2022) with the query sequences indicated in the corresponding figure or table legend using default parameters unless indicated otherwise. Multiple alignments were generated using the Clustal Omega program of Uniprot (https://www.uniprot.org/help/sequence-alignments; last accessed 26 April 2022). The frequency of exon usage or skipping and the frequency of splicing variants were calculated as reported elsewhere [31] with some modifications described in detail in Supplemental Material. For the analysis of terminator regions, cleavage sites were identified by visual inspection in EST sequences. It was then scored how often each site was used and the frequency was calculated as a percentage.

### 2.3. Cell Culture

Pooled HUVECs (C-12208, lot 447Z015; Promocell, Heidelberg, Germany) were cultivated in endothelial cell growth medium MV2 supplemented with 5% (*v*/*v*) fetal bovine serum, 5 ng/mL epidermal growth factor, 10 ng/mL basic fibroblast growth factor, 20 ng/mL insulin-like growth factor-1, 0.5 ng/mL vascular endothelial growth factor 165, 1 µg/mL ascorbic acid, and 0.2 µg/mL hydrocortisone (Promocell, Heidelberg, Germany). HSVSMCs were isolated from surplus vein tissue from consenting subjects undergoing coronary artery bypass graft surgery as previously described [32] and cultivated in smooth muscle cell growth medium 2 supplemented with 5% (*v*/*v*) fetal bovine serum, 0.5 ng/mL epidermal growth factor, 2 ng/mL basic fibroblast growth factor, and 5 µg/mL insulin (Promocell, Heidelberg, Germany). SMC integrity was verified by the presence of SMC markers myosin heavy chain and α-actin and the absence of the endothelial marker PECAM1 (CD31). Cultures were maintained at 37 °C in a humidified atmosphere containing 5% (*v*/*v*) CO_2_.

### 2.4. Isolation of Genomic DNA

HUVECs were lysed with TRIzol^®^ Reagent (ThermoFisher Scientific, Loughborough, UK) as per the manufacturer’s instructions. Briefly, the lysate was mixed with chloroform and centrifuged at 12,000× *g* for 15 min at 4 °C. The aqueous phase was discarded, and the DNA was precipitated with ethanol by centrifugation at 2000× *g* for 5 min at 4 °C. The DNA pellet was resuspended in 0.1 M sodium citrate in 10% (*v*/*v*) ethanol, pH 8.5, and centrifuged again after 30 min. The washing step was repeated and the pellet was resuspended in 70% (*v*/*v*) ethanol for 10–20 min. The DNA was centrifuged again at 2000× *g* for 5 min at 4 °C and the pellet allowed to air dry. The DNA was redissolved in pre-warmed (70 °C) TE buffer (10 mM Tris, 1 mM EDTA pH 8.0). The concentration and quality of the DNA were assessed with a NanoDrop 1000 spectrophotometer (ThermoFisher Scientific, Loughborough, UK).

### 2.5. Isolation of RNA and cDNA Synthesis

Total RNA was isolated using an Ambion^®^ mirVana™ miRNA Isolation Kit (ThermoFisher Scientific, Loughborough, UK) following the manufacturer’s instructions. The concentration and quality of the RNA samples were assessed with a NanoDrop 1000 spectrophotometer (ThermoFisher Scientific, Loughborough, UK). The total RNA (1 µg) was used for cDNA synthesis using a GoScript^TM^ Reverse Transcriptase kit from Promega (Southampton, UK), following the manufacturer’s instructions.

### 2.6. PCR, DNA Extraction and DNA Sequencing

Aliquots (1 µL) of cDNA were used as a template for standard 25 µL PCR reactions with primer pairs designed to explore alternatively spliced exons of *PPP1R12A*. Prior to being used on cDNA, primers were tested and PCR conditions optimized either on genomic DNA or on a plasmid containing the coding region of the full-length variant of *PPP1R12A* (IMAGE:40008469, GenBank: BC111752.1) as a template. Primer pairs were designed to produce amplicons of 200–800 bp. The primer pairs and reaction conditions are shown in Tables S1 and S2. Primer positions are shown in Figure S1. Negative control reactions used no template or a mock reverse transcriptase reaction as a template. The following general protocol was used: initial denaturation at 94 °C for 2 min; denaturation at 94 °C for 30 s, annealing at the optimized temperature for 30 s, elongation at 68 °C for 1 min per kbp, 35 or 42 cycles; final extension for 5 min at 68 °C. OneTaq^®^ DNA polymerase (New England Biolabs, Herts, UK) and its accompanying buffer were used. PCR reactions were run in an Applied Biosystems^TM^ Verity^TM^ 96-Well Thermal Cycler (ThermoFisher Scientific, Loughborough, UK).

The sizes of the amplicons obtained from the reactions were assessed using agarose gel electrophoresis and PCRBIO Ladder IV (PCR Biosystem Ltd., London, UK), and 1 kb Ladder and 100 bp Ladder (New England Biolabs, Ipswich, USA). The reactions were mixed with loading buffer (30% glycerol, 0.1% bromophenol blue) and 40% of the reaction was separated by gel electrophoresis on 1.5% agarose gels stained with ethidium bromide. The products were visualized and documented using a VersaDoc 1000 (Bio-Rad Laboratories, Watford, UK). The PCR products were extracted from the agarose gels using a NucleoSpin gel and PCR clean-up kit (Macherey-Nagel, Dueren, Germany). The identities of all PCR products were verified by sequencing (Eurofins Genomics, Cologne, Germany) with one of the primers used for amplification followed by sequence alignment using the nucleotide Blast tool at https://blast.ncbi.nlm.nih.gov/Blast.cgi (last accessed 22 May 2022).

## 3. Results

### 3.1. Transcription of PPP1R12A Is Driven by Three Promoter Regions

**Figure 1:**
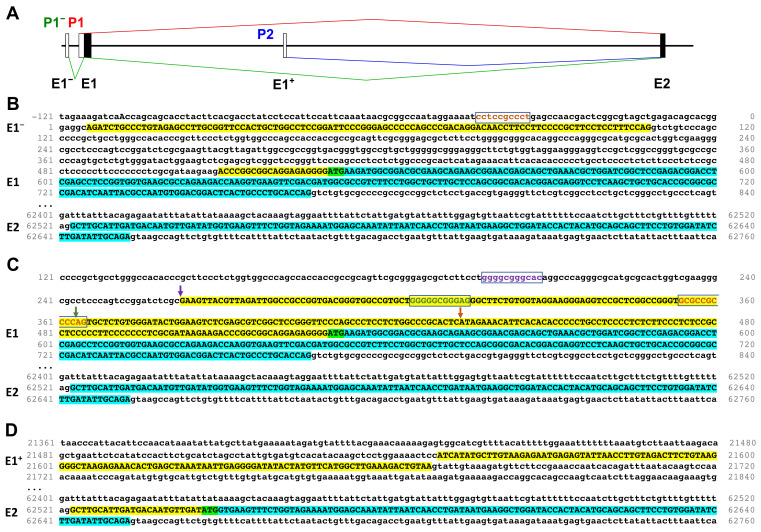
Predicted transcription start regions of *PPP1R12A*. (**A**) Schematic representation of the transcription start regions (P) based on Ensembl annotation. Distances between exons (E) are depicted at scale, except between E1^−^ and E1 for clarity. Black and white boxes represent translated or untranslated regions, respectively. Splicing arising from each promoter is indicated in a different color. (**B**) Transcription start at the P1^−^ region as per transcripts PPP1R12A-201 and -202. (**C**) Transcription starts at the P1 region as per transcripts PPP1R12A-203 and -208. (**D**) Transcription start at the P2 region as per transcript PPP1R12A-204. Transcribed DNA appears in upper case, of which translated DNA is highlighted in turquoise and untranslated DNA in yellow. Exon numbers are indicated on the left hand side. Predicted start codons are highlighted in green. Predicted Sp1 binding sites are framed and in colored fonts. Arrows in B indicate transcription start sites and are colored in the same scheme as the Sp1 binding site immediately upstream. DNA numbering is as per transcript PPP1R12A-202.

Since the Ensembl database suggests the existence of two transcription initiation sites for *PPP1R12A* in addition to the one reported by Machida et al. [21] (Figure 1A), we sought to obtain a clear picture of the promoter regions by examining information in the EST database. A BLASTn search with the UTR of E1 (267 bp) revealed a large number of EST clones (141) in support of the previously reported promoter region upstream of E1, which we name P1, acknowledging its frequent usage (Figure S2A). Three clusters of transcripts starting 96, 166, and 267 bp upstream of the start codon indicate the presence of three alternative transcription start sites in the P1 region (Figure S2A and Figure 1B).

Transcripts PPP1R12A-201 and -202 start upstream of E1. This exon, which we denominate E1^−^ (in order to preserve the exon nomenclature commonly used in the literature) is spliced into E1, leaving out a short (401 bp) intron and using the same translation start as transcripts PPP1R12A-203 and -208 (Figure 1C). A BLASTn search with E1^−^ plus 20 bp of the E1 UTR showed three clones documenting the existence of an infrequently (<0.1%) used promoter upstream of P1 (Figure S2B,C) that we call P1^−^. Of note, the region comprising P1^−^ and P1 overlaps with transcripts of the antisense gene *PPP1R12A-AS1* that can be distinguished by their opposite orientation. The P1^−^–P1 region is GC-rich and harbors an accumulation of regulatory elements (Ensembl Genome Browser). Sp1 has been shown to bind to this GC-rich promoter region and two Sp1 binding sites were identified [21]. We scanned the region with the ALGGEN-PROMO website for Sp1 sites and identified a total of four sites with a percentage of dissimilarity below 5%, two in addition to the ones previously recognized by Machida et al. [21]. These sites are each placed approximately 40–60 bases upstream of one of the transcription start sites in the P1^−^–P1 region (Figure 1B,C).

Transcript PPP1R12A-204 indicates a transcription start within intron 1, approximately 21 kb downstream of E1. This transcript includes a non-coding exon (that we name E1^+^) that splices into E2, shifting the start codon 261 bases downstream of the common start and producing a predicted protein lacking the first 87 residues of the full-length protein (Figure 1D). We name this predicted protein isoform ∆N. A BLASTn search with the sequences corresponding to E1^+^ and E2 revealed only two EST clones supporting the existence of an infrequently used promoter region that we call P2 (Figure S2D). Unlike the P1^−^–P1 region, the DNA immediately upstream of E1^+^ is not GC-rich.

### 3.2. Alternative Splicing of Coding Exons Gives Rise to 11 Protein-Coding PPP1R12A Transcripts

The Ensembl database collates the sequence and expression information of *PPP1R12A* into 23 transcripts distributed into three types: protein-coding, nonsense-mediated decay, and retained intron (Table S3). Four transcripts predict exons not annotated in GenBank within introns 2, 9, 20, and 22 that we name E2^+^, E9^+^, E20^+^, and E22^+^, in order to preserve the exon nomenclature commonly used in the literature (Figure S1). Transcripts including E20^+^ (PPP1R12A-217) and E22^+^ (PPP1R12A-205) are annotated as nonsense-mediated decay, whereas transcripts including E2^+^ (PPP1R12A-209) and E9^+^ (PPP1R12A-223) are annotated as protein coding. However, careful inspection shows that PPP1R12A-209 is more likely a nonsense-mediated decay transcript. Transcripts using E2^+^, E20^+^, and E22^+^ are very rare (Figure S3).

**Figure 2:**
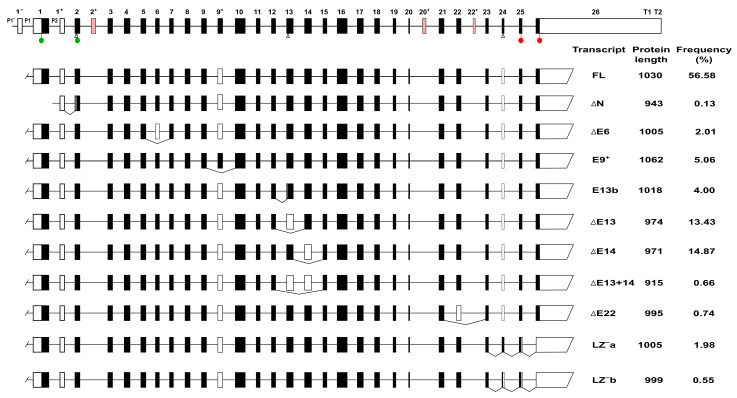
Transcription variants of *PPP1R12A* and their frequency. The top is a schematic representation of the *PPP1R12A* gene based on our analysis of data from Ensembl and GenBank. Only exons are depicted at scale. White boxes represent UTRs or spliced out exons, black boxes represent translated regions. Color boxes are exons found in nonsense-mediated decay transcripts. Triangles below E2, E13, and E24 indicate alternative acceptor splice sites. Green and red circles indicate translation start and stops, respectively. P and T denote promoter and terminator regions. Transcripts are truncated at both ends because there is insufficient information to assign particular promoters or terminators to specific transcripts. The scheme does not take into account possible combinations of alternatively spliced exons, for which there is no support. Most protein lengths are predicted. Transcript frequency was calculated from combined RNAseq data from AceView, GTEx, and GenBank. Splicing in of E9^+^ is only recorded in GenBank; therefore, the frequency of this variant was calculated relative to the GenBank data only.

**Figure 3:**
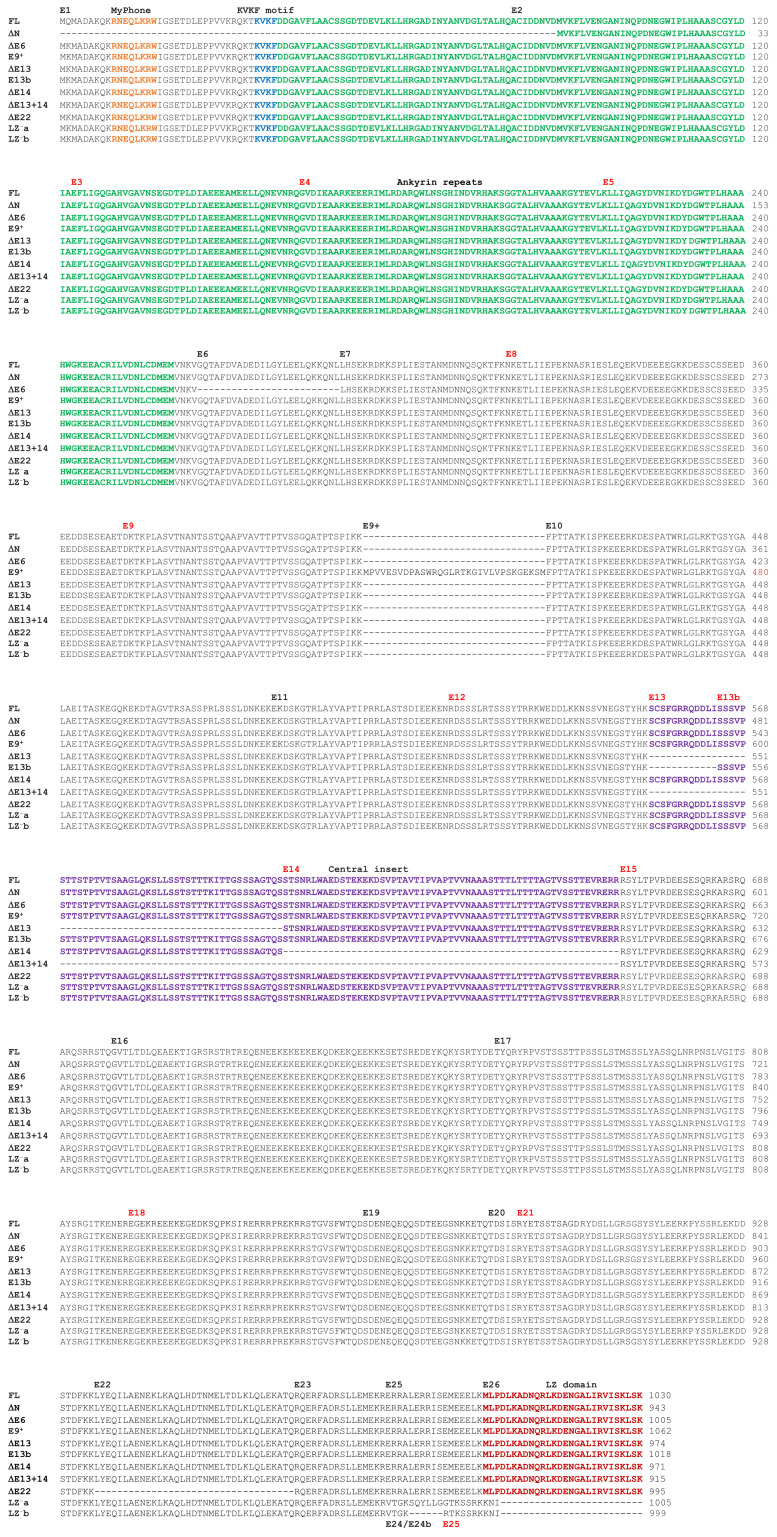
Multiple alignment of predicted human MYPT1 protein isoforms. The alignment was constructed using the Clustal Omega program of Uniprot. Exon boundaries are indicated on top of the alignment except for the LZ^−^ variants, which are at the bottom. Phase 0 exons (between codons) are labelled in black, and phase 1 or 2 exons (after the first or second base of a codon) in red. Domains and motifs are shown in different colors.

Taking into consideration only the protein coding transcripts in Ensembl, we identified 11, each encoding a predicted MYPT1 isoform. The exon composition of each transcript and their proposed names are shown in Figure 2. A multiple alignment of all the predicted isoforms is shown in Figure 3. The information available in GenBank does not support the presence of variants simultaneously carrying more than one alternatively spliced coding exon; however, that information is restricted to just 14 mRNA or cDNA sequences >1 kb in length along the coding region of *PPP1R12A*. These include complete coding regions for FL, ∆E13, ∆E22, and ∆N, and almost complete for ∆E14. No mRNA/cDNA sequences cover the ∆E6, E9^+^, ∆E13+14, LZ^−^a, and LZ^−^b variants. There is evidence of a transcription start at E1^−^ in the FL and ∆E22 variants, but the use of the P1^−^ region by other transcripts cannot be ruled out.

We next sought to revise the support for transcription variants of *PPP1R12A* involving coding exons E6, E9^+^, E13, E14, E22, and E24, most of which have not received attention before. In addition to exploring the EST database (Figure S4), we perused RNAseq information from GenBank, AceView, and GTEx to calculate the frequency of alternative splicing involving each of those six exons and the frequency of each of the 11 transcript variants (Figure 2 and Supplementary Spreadsheet S1).

The FL variant contains all coding exons, except E9^+^ and E24, is considered the canonical form and is the most abundant variant (56.6%), whereas ∆N is the least abundant variant (0.13%). Splicing variants of E6, E9^+^, E13, E14, and E22 all preserve the reading frame down to the stop codon in E26. E6 is spliced out in transcript PPP1R12A-216, resulting in the loss of a stretch of 25 amino acids. The splicing out of E6 occurs in 2% of transcripts on average. The inclusion of E9^+^ (transcript PPP1R123A-223) is predicted to result in a protein that is 32 amino acids longer than the FL variant. The E9^+^ variant is not supported by EST or mRNA sequences; however, there is clear support by RNAseq data, although it is captured only in the GenBank dataset. E9^+^ might account for 5% of variants.

E13 is spliced out in transcript PPP1R12A-213, resulting in a predicted protein that lacks a central stretch of 56 amino acids. This variant is relatively common (13.4%). Transcript PPP1R12A-221 contains a shorter variant of E13 (that we call E13b) that results from the usage of an alternative splicing acceptor site 36 bp into E13. The predicted protein lacks 12 amino acids in its central region. E13b accounts on average for 4.0% of variants. In transcript PPP1R12A-208, E14 is spliced out resulting in a predicted protein that lacks a central stretch of 59 amino acids. At 14.9%, this variant is, on average, slightly more common than ∆E13. In transcript PPP1R12A-206, both E13 and E14 are spliced out together, giving rise to a predicted protein that lacks a central stretch of 115 amino acids. This variant is very rare on average (0.66%).

E22 is spliced out in transcript PPP1R12A-202, resulting in a predicted protein that lacks 35 amino acids at its C-terminus. This variant is only supported by the mRNA sequence AF458589.1 (isolated from liver) and occurs in only 0.74% of transcripts. E24 is absent in most transcript variants of *PPP1R12A*. However, in two truncated transcripts, PPP1R12A-214 and PPP1R12A-222, this exon is spliced in, causing a frame shift that results in a premature stop and proteins lacking the leucine zipper domain (LZ^−^ isoforms). E24 can be spliced in in two different ways, to include either a 31-bp exon (transcript PPP1R12A-214) or a 13-bp exon (transcript PPP1R12A-222). Variants that splice E24 in are, on average, relatively uncommon: 1.98% the long (LZ^−^a) and 0.55% the short (LZ^−^b) variant.

### 3.3. Complex Profiles of PPP1R12A Transcription in Individual Cell Types of the Circulatory System Revealed by In Silico Analysis and RT-PCR

**Figure 4:**
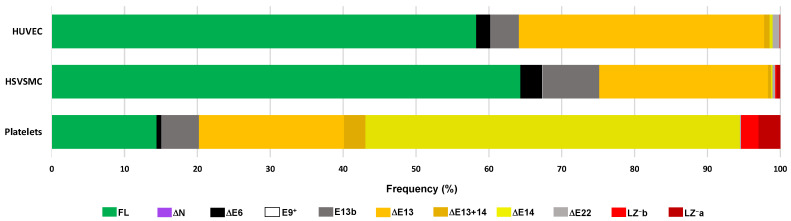
Frequency of *PPP1R12A* transcript variants in three individual cell types of the circulatory system. The frequency of each variant was calculated from RNAseq data collected from sets of GenBank BioProjects (see Supplementary Spreadsheet S2 for details).

To dissect the complexity of *PPP1R12A* expression in the circulatory system, we focused our attention on three cell types where MYPT1 is known to play important roles: endothelial cells (represented by HUVECs) [7], smooth muscle cells (represented by HSVSMCs) [19], and platelets [26,27]. We used a two-pronged approach consisting of the examination of RNAseq data (Figure 4 and Supplementary Spreadsheet S2) followed by verification and complementation by RT-PCR on cDNA prepared from cultured HUVECs and HSVSMCs. Prior to their use on cDNA, primers were tested either on genomic DNA or on a plasmid containing the coding region of the FL MYPT1 isoform (Figure S5).

**Figure 5:**
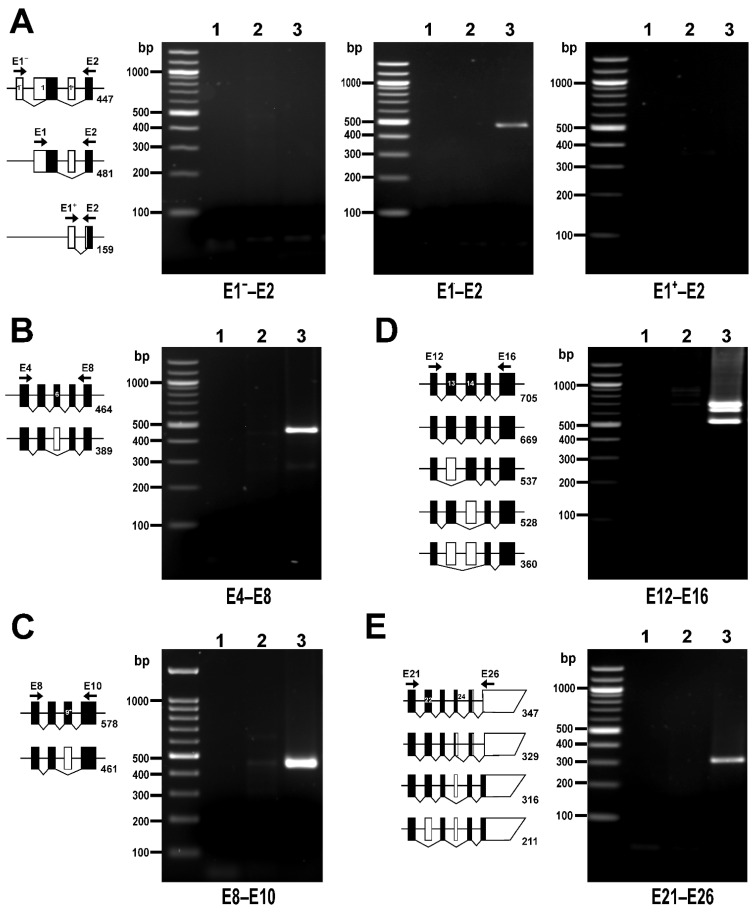
*PPP1R12A* transcripts in HUVECs detected by RT-PCR. The diagrams accompanying each panel depict the expected size of PCR products corresponding to all possible alternatively spliced variants sorted by size. White and black boxes represent untranslated and translated exons, respectively. Exons are depicted at scale. Position of primer pairs is indicated schematically. (**A**) PCR reactions targeting alternative transcription start sites. A transcription start on E1 is the only site used by HUVECs. (**B**) PCR reaction targeting E6 splicing variants. (**C**) PCR reaction targeting E9^+^ splicing variants. (**D**) PCR reaction targeting E13 and E14 splicing variants. (**E**) PCR reaction targeting E22 and E24 splicing variants. PCR reactions were run using no template (lane 1), a negative control for reverse transcriptase (lane 2), or cDNA (lane 3) as templates. Primer sequences and PCR conditions are shown in Table S2. In total, 40% of each 25 µL reaction was loaded on 1.5% agarose gels.

**Figure 6:**
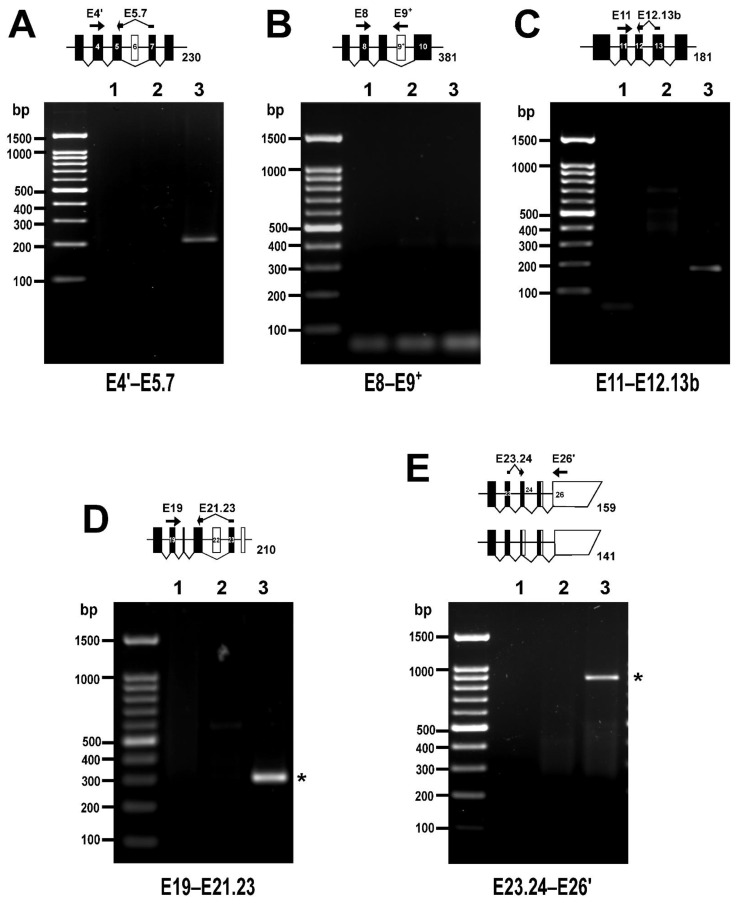
Rare *PPP1R12A* transcripts in HUVEC detected by RT-PCR. The diagrams accompanying each panel depict the expected size of PCR products corresponding to the rare alternatively spliced variants if present. White and black boxes represent untranslated and translated exons, respectively. Exons are depicted at scale. Position of primer pairs is indicated schematically. (**A**) PCR reaction targeting the ∆E6 splicing variant. (**B**) PCR reaction targeting the E9^+^ splicing variant. (**C**) PCR reaction targeting the E13b splicing variant. (**D**) PCR reaction targeting the ∆E22 splicing variant. (**E**) PCR reaction targeting LZ^−^ splicing variants. Asterisks (*) indicate unexpected PCR products. PCR reactions were run using no template (lane 1), a negative control for reverse transcriptase (lane 2), or cDNA (lane 3) as templates. Primer sequences and PCR conditions are shown in Table S2. In total, 40% of each 25 µL reaction was loaded on 1.5% agarose gels.

The analysis of the RNAseq data showed that HUVECs express two main transcriptional variants, FL (58.3%) and ∆E13 (33.7%), followed by the less abundant E13b (3.9%) and ∆E6 (1.9%). All other variants are expressed at very low levels (<1%, ∆E14, ∆E13+14, ∆E22, LZ^−^) or are undetectable (Figure 4). This transcriptional profile was confirmed by RT-PCR analysis (Figure 5 and Figure 6). PCR reactions addressing the three transcription initiation sites indicate that the transcription start on E1 is the only one used by HUVECs (Figure 5A). The PCR reaction targeting the alternative splicing of E6 yielded only the band corresponding to E6in variants (Figure 5B), whereas the PCR reaction targeting the alternative splicing of E9^+^ yielded only the band corresponding to E9^+^out variants (Figure 5C). The PCR reaction targeting the splicing variants of E13 and E14 yielded three bands (Figure 5D). The top band, approximately 700 bp, matches in size with the more abundant variants, including E13 and E14, and was verified by sequencing. The bottom band, slightly above 500 bp, is compatible with both ∆E13 and ∆E14. The sequencing of this amplicon ultimately confirmed that it corresponds to ∆E13. The middle band, approximately 650 bp, is compatible with the E13b splicing variant. However, sequencing of this amplicon returned a chromatogram compatible with a mix of E13in and E13out PCR products (Figure S6A). This 650 bp band probably arose by a hybridization of the top and bottom PCR products, as confirmed in a PCR reaction using a mixture of plasmids containing full-length and E13out MYPT1 sequences (Figure S6B). Figure 5E shows the result of the PCR reaction targeting the alternative splicing of E22 and E24. The single band slightly above 300 bp was confirmed by sequencing to correspond to an E22in/E24out transcript.

In order to capture very rare variants of *PPP1R12A,* a second set of primer pairs was designed in which one of the primers annealed either to one exon–exon boundary (to target exon-out variants) or to an exon normally not spliced in (to target exon-in variants) and increased the number of cycles (Table S2). PCR reactions using a reverse primer spanning the E5–E7 boundary yielded a relatively faint band of the expected size, confirming the presence of ∆E6 (Figure 6A). In contrast, PCR reactions using a reverse primer annealing on E9^+^ were negative, supporting the absence of an E9^+^ variant (Figure 6B). The splicing of the E13b variant of exon 13 was addressed with a reverse primer spanning the E13b-E12 boundary and the PCR reactions yielded a faint band of the expected size, confirming expression of this variant (Figure 6C). To explore the expression of ∆E22, a reverse primer spanning the E23–E21 boundary was used. This resulted in an amplicon of 300 bp, which was significantly larger than the expected 210 bp (Figure 6D). An inspection of the amplicon sequence revealed that the 3′ end of the reverse primer had annealed to and amplified through E22 and was therefore considered inconclusive. Finally, the expression of LZ^−^ variants was addressed with a forward primer spanning the E23–E24 boundary that should reveal either of the variants of this exon (Figure 6E). The PCR reactions yielded an unexpected 900-bp product that contained intronic sequences and may correspond to the retained intron transcript PPP1R12A-210.

Similar analyses were performed in HSVSMCs, where RNAseq data showed that, similar to HUVECs, these cells express two main variants, FL (64.3%) and ∆E13 (23.1%), followed by E13b (7.8%) and ∆E6 (3.0%); all other variants, including E9^+^, are expressed at very low levels (<1%) (Figure 4). RT-PCR analysis (Figures S7 and S8) confirmed that the predominant transcripts in these cells are the FL and ∆E13 variants. Variants ∆E6 and E13b were detected with reactions using exon-spanning primers, the E9^+^ variant was not detected, and reactions for the ∆E22 variant produced an inconclusive result as in HUVECs. The PCR reactions for LZ^−^ variants yielded three products: a 900-bp-long product, an intermediate > 500-bp product, and a very faint band slightly above 150 bp. The 900-bp product contained an intronic sequence and may correspond to the retained intron transcript PPP1R12A-210 as found in HUVECs. The >150 bp amplicon was confirmed to correspond to the LZ^−^a variant. Sequencing of the >500 bp amplicon returned a chromatogram compatible with a mix of the 900-bp and >150-bp PCR products (Figure S9A), suggesting that it resulted from a hybridization of the top and bottom PCR products, as confirmed by PCR reactions using the >500-bp amplicon as a template (Figure S9B).

Although platelets contain small amounts of residual mRNA, RT-PCR is not typically used to investigate gene expression in these cells; however, RNAseq is increasingly being used. RNAseq data from platelets showed that the predominant variants are those lacking the CI, accounting together for almost 75% of transcripts (51.4% ∆E14, 19.9% ∆E13, 3.0% ∆E13+14), followed by the FL variant (14.4%). LZ^−^ variants combined made up 5.4% of transcripts, with all other variants, including E9^+^, being expressed at very low levels (<1%) (Figure 4).

### 3.4. Complex Profiles of PPP1R12A Transcription across Human Tissues and Organs Revealed by In Silico Analysis of RNAseq Data

**Figure 7:**
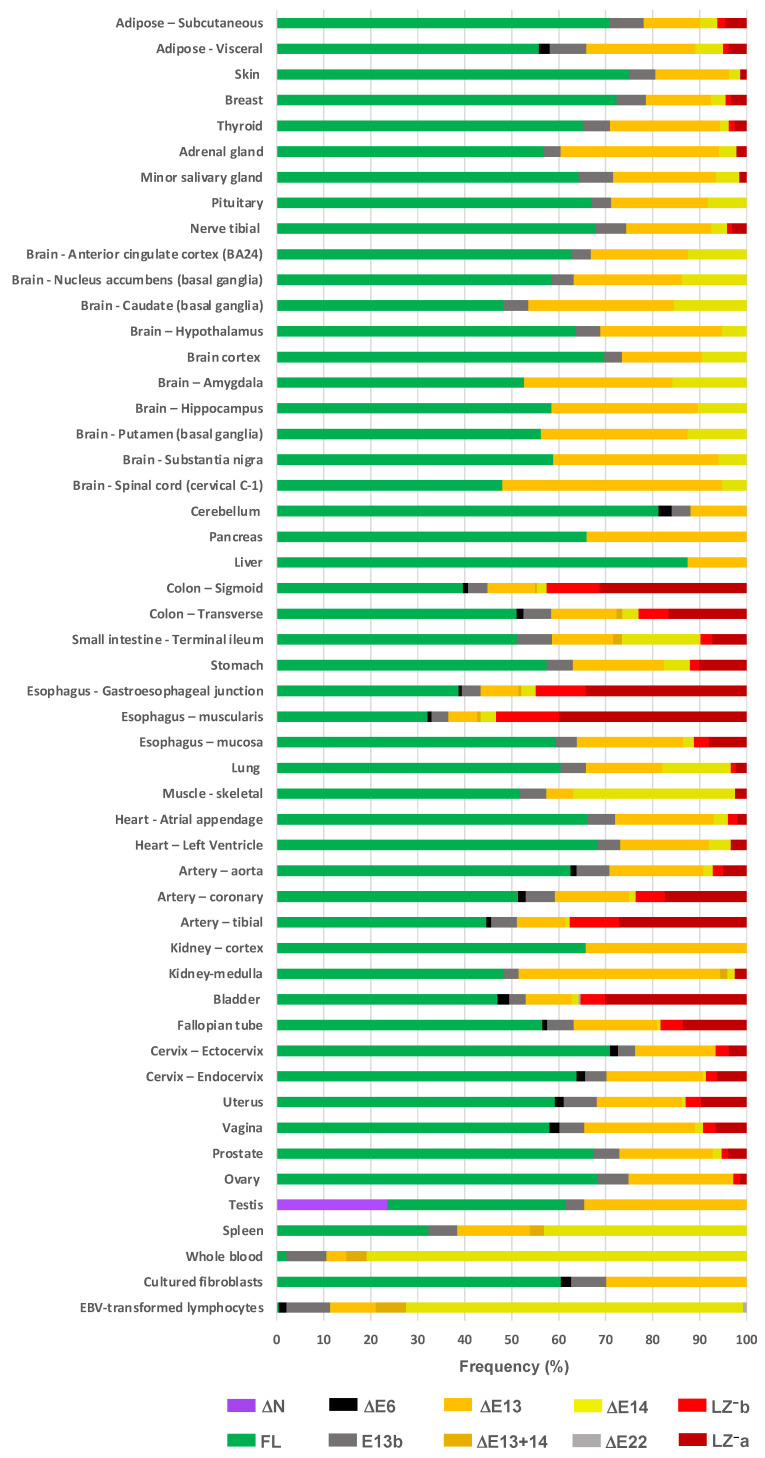
Frequency of *PPP1R12A* transcript variants in tissues, organs, and individual cell types. The frequency of each variant was calculated from RNAseq data from the GTEx portal (see Supplementary Spreadsheet S3 for details). The E9^+^ variant is not included due to it not been considered by GTEx. Tissues and organs have been grouped by body systems.

The GTEx database constitutes an excellent resource to address the transcriptional landscape of *PPP1R12A* across almost all tissues and organs. We therefore used RNAseq data to calculate the frequency of each transcription variant in each tissue, organ, and cell type sample (Figure 7 and Supplementary Spreadsheet S3). We observed that the FL variant is the most frequent variant in most tissues and organs. Exceptions are the esophagus muscularis (32.1%), spleen (32.3%), and whole blood, where this variant is rare (2.1%).

Variants involving the CI region (E13 and E14) follow the FL variant in relative frequency. The ∆E13 variant is ubiquitous, particularly frequent (>30%) in brain, pancreas, adrenal gland, kidney, and testis, and less frequent in skeletal muscle (5.7%) and whole blood (4.3%). E13b is documented in most organs; it is relatively more abundant in arteries, intestine, genitourinary tract, adipose tissue, and whole blood and less abundant in the brain. The ∆E14 variant is undetectable in some organs but is notably abundant in blood (81%), spleen (43%), skeletal muscle (34%), small intestine (17%), and some parts of the brain. ∆E13+14 is present in only a few organs, including spleen and whole blood—where it is relatively more abundant (3–4%), kidney medulla, esophagus, and intestine.

Regarding LZ^−^ variants, usually both are present in the same organ. They are relatively abundant in organs rich in smooth muscle such as arteries, bladder, esophagus, stomach, intestine, uterus, and oviducts. Both variants combined range between 10 and 52% in frequency. They are less frequent in organs such as prostate, cardiac and skeletal muscle, adipose tissue, and breast and are absent from blood, spleen, brain, testis, liver, and pancreas.

∆E6 is documented in approximately one third of tissues but is infrequent (<3%). ∆E22 is documented in bladder and is very rare (<0.5%). Notably, the ∆N transcript variant appears to be expressed exclusively in the testis, where it accounts for 24% of *PPP1R12A* transcripts. The GTEx study includes two cell lines; in cultured fibroblasts, the main variants are the FL (60%) followed by the ∆E13 (30%) variant, whereas in Epstein–Barr virus (EBV), transformed lymphocytes’ CI variants (mainly ∆E14) account for almost 90% of *PPP1R12A* transcripts.

### 3.5. Termination of PPP1R12A Transcription Occurs at Two Regions

**Figure 8:**
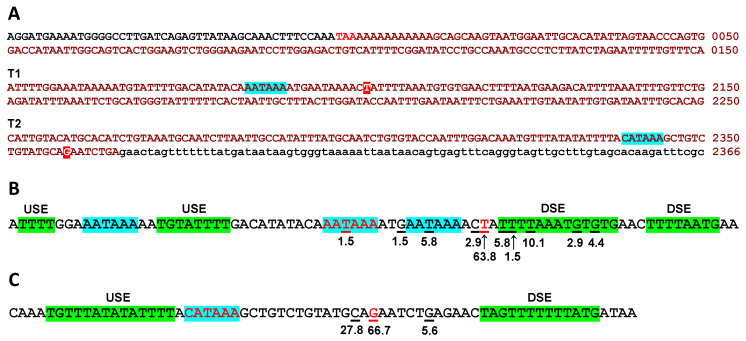
Predicted terminator regions of *PPP1R12A*. (**A**) Positions of the terminators relative to the stop codon on E26. The coding sequence is shown in black, stop codon in red, 3′-UTR sequences in ochre upper-case letters (see Figure S1 for the complete E26 sequence). The genomic sequence further downstream appears in lower-case letters. The most likely polyadenylation signals are highlighted in turquoise and the most frequent cleavage sites in red. (**B**) Analysis of T1 and (**C**) of T2. Putative polyadenylation signals are highlighted in turquoise and putative upstream (USE) and downstream (DSE) sequence elements in green. Cleavage sites identified in EST sequences are underlined and their relative frequencies (%) are indicated. The most likely polyadenylation signals and the most frequent cleavage sites appear in red characters.

The termination region of *PPP1R12A* has not been examined previously. To determine this region, a BLASTn search was performed with a 2418-bp cDNA sequence encompassing the last 52 bp of the coding sequence followed by the 3′-UTR as per Ensembl transcript PPP1R12A-201. This search revealed an accumulation of ESTs that terminate sharply approximately 2100 bp downstream of the stop codon, suggesting the presence of a terminator in that area (T1), while several EST clones continue past T1, supporting the presence of another termination region (T2) approximately 260 bp further downstream (Figure S10 and Figure 8A). No EST clones were found extending beyond the PPP1R12A-201 sequence in a search of genomic DNA sequences further downstream of the T2 region.

An inspection of the DNA sequences of 69 informative clones out of 71 ending at the T1 area (Table S4) showed that most of them terminate at thymine 2102 (counting from the last base of the stop codon) (63.8%) or in close vicinity (20.3%) (Figure 8B). Three canonical polyadenylation signals are identifiable in this region, of which the central one appears to be the most commonly used as it is placed 11–17 bp upstream of a cluster of cleavage sites around thymine 2102. A T-rich upstream sequence element (USE) can be identified 10 bp upstream of the polyadenylation signal and two T/GT rich downstream sequence elements (DSE) immediately after the most frequent cleavage site [33]. The two other potential polyadenylation sites appear to be less frequently used but may account for cleavage sites upstream (8.8%) and downstream (7.3%) of the cluster of frequent cleavage sites mentioned above.

An inspection of the DNA sequences of 18 informative clones out of 20 ending at the T2 area (Table S5) showed that 66.7% terminate at guanine 2359, with the rest in close vicinity (Figure 8C). A non-canonical polyadenylation signal (CATAAA) can be identified 15 bp upstream of the most frequent cleavage site and is immediately preceded by a T-rich upstream USE. A T-rich DSE is placed 11 bp downstream of the most frequent cleavage site.

Finally, the stop codon of *PPP1R12A* is embedded in an AATAAA sequence that could function as a polyadenylation signal (Figure 8A), and numerous ESTs end at or just after the stop codon. The most likely explanation for these is that the polyT primer used to construct the cDNA libraries annealed to the stretch of adenines that follows the stop codon.

## 4. Discussion

The presence of alternative splicing variants of the gene encoding MYPT1 was recognized in early studies, and the functional relevance of a small number of variants has been investigated extensively [2,10,13,14,19]. However, previous studies have been restricted to few organs and tissues and do not reflect the complexity of the transcriptional capabilities of *PPP1R12A*, which is important to understand the specific cellular roles of MYPT1 isoforms. To begin filling these knowledge gaps, we have undertaken a comprehensive examination of the transcriptional landscape of the *PPP1R12A* gene, with a focus on protein-coding transcripts. Our study revealed a previously unrecognized complex transcriptional repertoire for the human *PPP1R12A* gene. We establish a total of 32 exons, 29 of which are capable of encoding a minimum of 11 predicted protein variants expressed in different proportions across pure cell types and organs. Three exons are present only in nonsense-mediated decay transcripts. An analysis of RNAseq data allowed us to generate quantitative transcript profiles of *PPP1R12A* in HUVECs, HSVSMCs, and platelets, with up to 10 transcripts expressed with unique patterns of frequencies. Our findings place us in a better position to design functional studies targeting MYPT1 isoforms.

RT-PCR, both conventional and quantitative, has been commonly used to determine the presence and relative abundance of the LZ^−^ variant [12,13,15,16], and less frequently of CI variants [7,10,18,19]. While fit for their purpose, such approaches do not capture the complexity of the transcriptional status of *PPP1R12A*, particularly when investigating the function of MYPT1 in a particular cell type. Our conventional RT-PCR approach reveals some caveats when contemplating using PCR for a comprehensive analysis of *PPP1R12A* variants, including the need to design and test primers and optimize reaction conditions for a number of primer sets, as well as the risk of missing variants that are expressed at very low levels. We propose taking into consideration an approach based on RNAseq data as a tool for gaining a comprehensive quantitative picture of *PPP1R12A* variants in a tissue or cell type of interest if suitable public data are available or can be generated.

MYPT1 plays important roles in the circulatory system. Its role as a key modulator of smooth muscle contractility is well established [4]. In the endothelium, MYPT1 is important for the maintenance of barrier function [7,22,23,24,25], whereas in platelets it regulates shape change, spreading, and thrombus stability [26,27]. The profiles of *PPP1R12A* transcripts in HUVECs and HSVSMCs are very similar, with a predominance of the FL variant followed by the ∆E13 variant. HSVSMCs express unexpectedly low levels of LZ^−^ variants. Studies on MYPT1 LZ^−^ variants in venous smooth muscle, where these variants are reported to be very abundant, have been carried out in rat tissue rather than in cultivated smooth muscle cells [11,13,19,34]. Smooth muscle cells undergo dedifferentiation when cultivated, which likely affected the levels of LZ^−^ variants. In platelets, LZ^−^ variants account for 5.4% of transcripts, suggesting that, similar to what has been described for smooth muscle, sensitivity to nitric oxide signaling might also play an important modulatory role in platelets.

According to our analyses, MYPT1 isoforms lacking the CI region are ubiquitous and relatively common. In most tissues and cell types, CI variants are found at various proportions, ∆E13 usually being the predominant CI variant. Notably, in hematopoietic cells (including platelets) and organs, the ∆E14 variant is not only the main CI variant, but the most frequent of all variants, clearly suggesting specific roles for ∆E14 in those cells. In spite of their relative abundance, CI variants have received little attention. ∆E13 and ∆E14, along with a E13b equivalent, and an even shorter version of E13 have been reported in rats [10,19]. Dirksen et al. (2000) used a PCR approach to determine the relative proportions of FL and CI variants in various rat organs [10]; these are in good agreement with our results using human RNAseq data. E13 and/or E14 variants have been annotated in various vertebrates, indicating that they are phylogenetically conserved. Very little is known about the consequences of CI deletions for MYPT1 function. In HeLa cells, where FL and ∆E13 are the main variants, the silencing of individual variants showed that both are important for the dephosphorylation of MLC and regulation of the actin cytoskeleton architecture, with only subtle differences between variants [5]. One obvious consequence would be the removal of binding sites for interacting proteins, as is the case with ∆E13 reportedly showing a low binding affinity for radixin [7]. Another possible consequence is an alteration in susceptibility to regulation of MLCP activity by phosphorylation of critical residues situated in the vicinity of the CI. Although not fully comparable to mammalian CI variants, a lower rate, but not extent, of PKG-mediated phosphorylation in vitro has been reported in the ∆E12 chicken variant [20].

The LZ^−^ isoforms of MYPT1 have been extensively studied, predominantly using avian and rodent models, and more recently this knowledge has been transposed to humans, where E24 is not annotated in GenBank. Dordea et al. (2013) [15] and Lartey et al. (2016) [16] used quantitative RT-PCR to demonstrate the LZ^−^ variant in placental and myometrial arteries and in uterine smooth muscle, respectively. In a recent extensive study, Oslin et al. (2022) [12] used a combination of genetic mouse models, human tissues, and public RNAseq data (including GTEx) to characterize the expression of the LZ^−^ variant. In the mouse, this variant is abundant in smooth muscle isolated from various organs, broadly matching the RNAseq pattern we observe in human tissues. However, the ratios of LZ^−^/LZ^+^ variants in isolated murine smooth muscle cannot be compared directly to the ratios calculated from the RNAseq data of human tissues and organs because these contain a mix of cell types each with its own pattern of *PPP1R12A* variants. This limitation is clearly illustrated in the GTEx dataset for esophagus, where the muscularis expresses more that 50% LZ^−^ variants whereas the mucosa expresses just above 10% (Figure 7), and has been recognized by others [12]. It is interesting to note that the existence of an LZ^−^ variant with a 13-bp version of E24 has not been previously detected. This shorter LZ^−^b variant is less frequent than the longer LZ^−^a variant but is expected to be functionally equivalent.

As well as refining our knowledge about CI and LZ variants, our analysis has consolidated the existence of a ∆E22 variant and uncovered two more, ∆E6 and E9^+^. These three variants bring about the deletion or addition of short stretches of amino acids (25–35 residues) and are infrequent, although we cannot rule out a higher relative abundance and a clear functional relevance in specific cell populations. Out of these three rare variants, E9^+^ seems to be phylogenetically conserved in some vertebrates, and the exon is annotated and supported by rat RNAseq data (GenBank).

In this study, we have extended our knowledge about the *PPP1R12A* promoter region by identifying a total of four predicted transcription initiation sites, each with an Sp1 binding site, one of them in a novel P1^−^ promoter region shortly upstream of the most frequently used P1 promoter region. The three transcription initiation sites in the P1 region appear to be used equally frequently, but it remains to be established why the site in the P1^−^ region is rarely used. Very little is known about the transcriptional regulation of *PPP1R12A*, but it is noteworthy that *PPP1R12A-AS1*, the gene that overlaps with the 5′ region of *PPP1R12A*, encodes an antisense transcript that functions as a translation up-regulator [35]. The novel ∆N variant of MYPT1 seems to arise from a transcription start located within I1 of *PPP1R12A* that reads into exon E1^+^. Although this variant is globally very rare, it accounts for about one fourth of *PPP1R12A* transcripts in testis, the only organ where it is expressed. What cell type(s) within the testis express(es) the ∆N variant and what role it plays will require investigation. A ∆N variant is not documented in rodents (GenBank), where MYPT1 has been widely researched. However, in *C. elegans,* a potential splice variant that lacks the first 37 residues of the MYPT1 ortholog functions normally in the spermatheca but not in the embryonic epidermis [36]. One potential consequence of the N-terminal deletion is the ablation of the ability of MYPT1 to bind PP1c, as three PP1c-binding regions would be missing: the K35VKF38 motif that appears to be essential for the interaction, the MyPhoNE motif, and the first ankyrin repeat [37,38]. In addition, nuclear localization signals identified in the N-terminal region are lost [39,40], likely resulting in a protein that is unable to translocate to the nucleus, where MYPT1 is known to regulate gene expression [1].

The termination of *PPP1R12A* transcription is apparently governed by two polyadenylation sites: a strong site supported by 80% of ESTs and controlled by a canonical polyadenylation signal, and a weak site supported by 22% of ESTs and controlled by an alternative signal. Multiple polyadenylation signals are not uncommon and in such cases the distal site more frequently uses a canonical signal and all other sites use non-canonical signals [41]. This is not the case for *PPP1R12A*; however, in line with published observations [41], we observed that the canonical signal is more efficiently processed than the non-canonical one. This may be due in part to a longer T-rich DSE associated with the canonical signal [42]. Meanwhile, the use of alternative termination sites with different polyadenylation rates is postulated as a mechanism to regulate the synthesis of specific mRNA forms and also affect protein expression [43], but this aspect remains to be investigated in *PPP12R12A*.

In summary, our study extends the knowledge about the complex patterns of transcription of *PPP1R12A* in cells of the circulatory system and across tissues. We expect this information will contribute to guide future studies on the specific roles of understudied MYPT1 isoforms in health and disease.

## Data Availability

Data related to *PPP1R12A* expression were retrieved from public databases and analyzed as indicated in the Materials and Methods section and in the Supplementary Spreadsheets 1, 2, and 3.

## Supplementary Materials

The following supporting information can be downloaded at: www.mdpi.com/article/10.3390/cells11152315/s1, Supplemental methods: Calculation of the frequency of splicing variants; Table S1: Oligonucleotide pairs used for control PCR reactions; Table S2: Oligonucleotide pairs used for RT-PCR reactions; Table S3: *PPP1R12A* transcripts and protein variants as annotated in Ensembl and GenBank; Table S4: Collection of EST clones supporting terminator region T1 approximately 2100 bp downstream of the stop codon; Table S5: Collection of EST clones supporting terminator region T2 approximately 2360 bp downstream of the stop codon. Figure S1: Exon composition of *PPP1R12A* and location of PCR primers used in this study. Figure S2: Results of BLASTn searches for ESTs in support of transcripts with non-canonical exons. Figure S3: Results of BLASTn searches for ESTs in the transcription start regions of *PPP1R12A*. Figure S4: Results of BLASTn searches for ESTs in support of alternatively spliced exons; Figure S5: Test PCR reactions of *PPP1R12A* primers; Figure S6: Hybridization of PCR products of reaction targeting E13 and E14 variants; Figure S7: *PPP1R12A* transcripts in HSVSMCs as determined by RT-PCR; Figure S8: Rare *PPP1R12A* transcripts in HSVSMCs as determined by RT-PCR; Figure S9. Hybridization of PCR products of the reaction targeting E24. Figure S10: Results of BLASTn search for ESTs in the 3′-UTR region of *PPP1R12A*. Supplementary Spreadsheet S1: EST, mRNA/cDNA and RNAseq data supporting PPP1R12A alternatively spliced variants; Supplementary Spreadsheet S2: Determination of the frequency of *PPP1R12A* variants in individual cell types; Supplementary Spreadsheet S3: *PPP1R12A* RNAseq data from the GTEx project.
