# Supplementary material for: Complex Transcriptional Profiles of the PPP1R12A Gene in Cells of the Circulatory System as Revealed by In Silico Analysis and Reverse Transcription PCR
Source: Cells. 2022 Jul 27;11(15):2315. doi: 10.3390/cells11152315 (PMC9367544; doi:10.3390/cells11152315)
Supplement: Supplementary file 1 [file cells-11-02315-s001.zip › Supplemental files/Supplemental Material-Proofs.pdf]

Supplemental Materials

Supplemental Methods

Calculation of the frequency of splicing variants

To account for the fact that in many cases the RNAseq reads are not distributed uniformly along the *PPP1R12A* cDNA, calculations were made relative to the regions where splicing events take place. Consider the following example where exon E_c can be alternatively spliced.

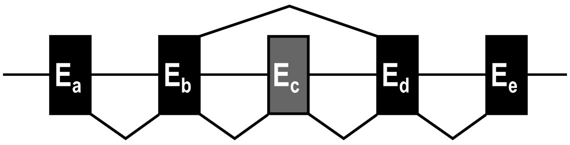

For each region, first the average number of reads spanning constitutive exon junctions was calculated using the following formula:

$$N = [E_aE_b + (E_bE_c + E_bE_d) + (E_cE_d + E_bE_d) + E_dE_e] / 4$$

where E_aE_b is the number of reads spanning exons E_a and E_b, and so on. Variations of the above formula were used for alternative splicing donor or acceptor sites and in regions involving splicing of more than one exon. The frequency (expressed as percentage) of skipping or splicing in of E_c is calculated as:

$$\%\text{Splicing out} = 100 * E_bE_d / N$$

$$\%\text{Splicing in} = 100 * [(E_bE_c + E_cE_d) / 2] / N$$

To calculate the frequency (expressed as percentage) of each variant we assumed only one exon is alternatively spliced in every variant, as there is no evidence of *PPP1R12A* variants resulting from simultaneous splicing of two or more exons other than E13+E14. The frequency of LZ^- variants was taken as the frequency of splicing in of E24 (LZ^-a) and E24b (LZ^-b). The frequency of all other (LZ^+) variants was calculated relative to (100 - LZ^-a - LZ^-b). For example, the frequency of the ΔE14 variant was calculated as:

$$\%\Delta E14 = \%\text{Splicing out E14} * (100 - LZ^-a - LZ^-b) / 100$$

Finally, the frequency of the FL variant was calculated as 100 minus the sum of frequencies of all other variants.

Supplemental Tables

Table S1. Oligonucleotide pairs used for control PCR reactions. Primer pairs are given as forward (top)-reverse (bottom). In the amplicon size column (g) indicates that the primers were tested using genomic DNA as template; (p) indicates that the primers were tested using a plasmid containing the *PPP1R12A* variant encoding the FL MYPT1 variant as a template. The position of the primer pairs and their respective amplicons are shown in Fig. S5A. Primers in blue characters were used exclusively for testing the other member of the pair and not further used in RT-PCR reactions. E19 and E26' were used in combination to test each other and subsequently in separate RT-PCR reactions. Annealing temperature and number of cycles for the PCR reactions are indicated. Where the PCR buffer departed from the standard this is also indicated.

Primer pair	Sequence (5'-3')	Amplicon size (bp)	PCR conditions
E1^{-}–E1	ATCTGCCCTGTAGAGCCTTG<br>TTCACCTGGTCTTCTGGCGC	632 (g)	55.4°C, 42x, GC-enriched buffer + DMSO
E1–E1	GGATACTGGAAGTCTCGAGCG<br>TTCACCTGGTCTTCTGGCGC	265 (g)	55.4°C, 42x, GC-enriched buffer + DMSO
E1^{+}–I1	AGGGGCTAAGAGAAACACTGA<br>CACAAACAGACAATGCACACA	158 (g)	58°C, 42x
E1'–E2	ATGAAATGGCGGACGCG<br>GGTATCCAGCCTTCATTATCAGG	326 (p)	60°C, 35x
E4–E8	CGGCATGCAAAATCTGGAGG<br>TCCTTCTTCTTCTTCATCAACC	465 (p)	52°C, 35x
E4'–E8	TACAGCACTTCACGTTGC<br>TCCTTCTTCTTCTTCATCAACC	445 (p)	61°C, 35x
E8–E10	AGACGTTGATTATTGAACCAGAG<br>GGGACTTGAAGCTGAACGTG	461 (p)	58°C, 35x
I9–E9^{+}	TGTTTGGTGGGGAAATCAGTAAA<br>CCTTACTGGGCACAAGAACAATG	523 (g)	58°C, 42x
E12–E16	AGGAGAAAATGGGAAGATGATC<br>CTCATCATACGTTCTGGAGTAC	705 (p)	56°C, 35x
E21–E26	AGAAAACCTTACAGCAGCAGG<br>TCAAGGCCCCATTTTCATCC	316 (p)	56°C, 35x
E19–E26'	CAGACACAGAAGAGGGATCCA<br>ATAACTCTGATCAAGGCCCC	463 (p)	56°C, 35x

Table S2. Oligonucleotide pairs used for RT-PCR reactions. Primer pairs are given as forward (top)-reverse (bottom). The position of the primer pairs and expected amplicons for each variant are shown in Figs. 5, 6, S7 and S8. In primers spanning exon boundaries the side of the upstream exon is underlined. Annealing temperature and number of cycles for the PCR reactions are indicated.

**Primer pair**	**Sequence (5'-3')**	**Variants (amplicon sizes in bp)**	**PCR conditions**
E1$^-$–E2	ATCTGCCCTGTAGAGCCTTG<br>TTCACTTGGTCTTCTGGCGC	E1$^-$ in (447)<br>E1$^-$ out (0)	55°C, 35x
E1–E2	GGATACTGGAAGTCTCGAGCG<br>TTCACTTGGTCTTCTGGCGC	E1 in (481)<br>E1 out (0)	56°C, 35x
E1$^+$–E2	AGGGGCTAAGAGAAACACTGA<br>GGTATCCAGCCTTCATTATCAGG	E1$^+$ in (159)<br>E1$^+$ out (0)	55°C, 35x
E4–E8	CGGCATGCAAAATCTGGAGG<br>TCCTTCTTCTTCTTCATCAACC	E6 in (465)<br>E6 out (389)	52°C, 35x
E8–E10	AGACGTTGATTATTGAACCAGAG<br>GGGACTTGAAGCTGAACGTG	E9$^+$ in (578)<br>E9$^+$ out (461)	58°C, 35x
E8–E9$^+$	AGACGTTGATTATTGAACCAGAG<br>CCTTACTGGGCACAAGAACAATG	E9$^+$ in (381)<br>E9$^+$ out (0)	58°C, 42x
E12–E16	AGGAGAAAATGGGAAGATGATC<br>CTCATCATACGTTCTGGAGTAC	E13 in–E14 in (705)<br>E13b in–E14 in (669)<br>E13 out–E14 in (537)<br>E13 in–E14 out (528)<br>E13 out–E14 out (360)	54°C, 35x
E21–E26	AGAAAACCTTACAGCAGCAGG<br>TCAAGGCCCCATTTTCATCC	E22 in–E24 in (347)<br>E22 in–E24b in (329)<br>E22 in–E24 out (316)<br>E22 out–E24 out (211)	58°C, 35x
E4'–E5.7	TACAGCACTTCACGTTGC<br>CCGTTTTTCACTATGGAGCACT	E6 in (0)<br>E6 out (211)	61°C, 42x
E11–E12.13b	TGCACCTACAATACCAAGACGA<br>GCTTGGAACACTAGAACTTTTATG	E13 in (0)<br>E13b in (181)	53°C, 42x
E19–E21.23	CAGACACAGAAGAGGGATCCA<br>CAGCAAATCTTTCTTGTCTCTTT	E22 in (0)<br>E22 out (210)	55°C, 42x
E23.24–E26'	GAAAAAAGGGTGACCGGCAAG<br>ATAACTCTGATCAAGGCCCC	E24 in (159)<br>E24b in (141)	56°C, 42x

Table S3. *PPP1R12A* transcripts and protein variants as annotated in Ensemble and GenBank. All transcripts are supported by EST and/or mRNA sequences and for the protein coding variants also by RNAseq data, with the exception of transcript PPP1R12A-223, which is only supported by RNAseq data. The variant considered canonical in Uniprot uses all coding exons except $E9^{+}$, E24 and exons only found in nonsense-mediated decay transcripts ($E2^{+}$, $E20^{+}$ and $E22^{+}$). Transcript PPP1R12A-209 is annotated as protein coding but is more likely a nonsense-mediated decay transcript. Most Ensemble transcripts are incomplete and have been extended as shown in Fig. 2 to calculate the predicted protein isoforms.

**Ensembl (GenBank) transcript**	**Type**	**Splicing**	**Protein isoform (predicted length) (GenBank isoform)**
PPP1R12A-201 (NM_001143885.2, variant 2)	Protein coding	Canonical, start at $E1^{-}$	FL (1030) (isoform a)
PPP1R12A-202 (NM_001244990.2, variant 4)	Protein coding	E22 out, start at $E1^{-}$	ΔE22 (995) (isoform c)
PPP1R12A-203 (NM_002480.3, variant 1)	Protein coding	Canonical, start at E1	FL (1030) (isoform a)
PPP1R12A-204 (NM_001143886.2, variant 3)	Protein coding	Start at $E1^{+}$	ΔN (943) (isoform b)
PPP1R12A-205	Nonsense-mediated decay	$E22^{+}$ in	
PPP1R12A-206	Protein coding	E13+E14 out	ΔE13+14 (915)
PPP1R12A-207	Retained intron		
PPP1R12A-208	Protein coding	E14 out	ΔE14 (971)
PPP1R12A-209	Protein coding (Nonsense-mediated decay)	$E2^{+}$ in	
PPP1R12A-210	Retained intron		
PPP1R12A-211	Retained intron		
PPP1R12A-212	Retained intron		
PPP1R12A-213 (NM_001244992.1, variant 5)	Protein coding	E13 out	ΔE13 (974) (isoform d)
PPP1R12A-214	Protein coding	E24 in (31 bp variant)	LZ^{-}a (1005)
PPP1R12A-215	Retained intron		
PPP1R12A-216	Protein coding	E6 out	ΔE6 (1005)
PPP1R12A-217	Nonsense-mediated decay	$E20^{+}$ in	
PPP1R12A-218	Retained intron		
PPP1R12A-219	Retained intron		
PPP1R12A-220	Retained intron		
PPP1R12A-221	Protein coding	Alternative splicing acceptor site in E13	E13b (1018)
PPP1R12A-222	Protein coding	E24 in (13 bp variant)	LZ^{-}b (999)
PPP1R12A-223	Protein coding	$E9^{+}$ in	E9+ (1062)

Table S4. Collection of EST clones supporting terminator region T1 approximately 2100 bp downstream of the stop codon. Accession numbers are hyperlinked to the respective GenBank submission. Sequences at the end of each clone were taken directly from the GenBank submission, note that most are in antisense direction. Cleavage sites are highlighted in turquoise unless reliable identification was not possible. *Clones without a clear polyA stretch but with annotation that an oligo dT was used in the cDNA synthesis. §Clones without a clear polyA stretch and no annotation about an oligo dT being used in the cDNA synthesis.

Accession number	Tissue or cell type	Sequence at end of clone
BU620191.1	Chondrosarcoma cell line	tttttttttt tttttttttaa tagttttatt cattttattt gtatatgtca
CA312676.1	Lung epithelial cells	tttttttttt ttttttttca cacatttaaa atagttttat tcattttatt
CN278743.1	Embryonic stem cell line	aaatgtattt tgacatatac caataaaatg aataaaacta aaa
BU677557.1	Lung epithelial cells	tttttttttt ttttttttag ttttattcat tttatttgta tatgtcaaaa tacattttta
CA775922.1	Pancreatic islet	tttttttagg tttattcatt ttatttgtat atgtcaaaat acatttttat
BQ477501.1	Insulinoma	tttttttttt tttttagttt tattcatttt atttgtatat gtcaaaatat
CA310990.1	Lung epithelial cells	tttttttttt ttttttttag ttttattcat tttatttgta tatgtcaaaa tacattttta
BM993875.1	Subchondral bone	tttttttttt ttttttttat agntttattc attttatttg tatatgtcaa
CN479410.1	Subchondral bone	tttttttttt ttttttttat agntttattc attttatttg tatatgtcaa
BM968622.1	Lung epithelial cells	tttttttttt ttttttttca cacatttaaa atagttttat tcattttatt
AW001851.1	Thymus	tttttttttt tcacatttaa aatagtttta ttcattttat ttgtatatgt
CA440121.1	Chondrosarcoma	tttttttttt ttttttttaa aatagtttta ttcattttat ttgtatatgt
AI828759.1	Endometrial adenocarcinoma	tttttttttt aaaatagttt tattcatttt atttgtatat gtcaaaatac
BF197980.1*	Skin	aaaatagttt tattcatttt atttgtatat gtcaaaatac atttttattt
AI342381.1	Uterus	tttttttttt atagttttat tcattttatt tgtatatgtc aaaatacatt
CD678763.1	Eye trabecular meshwork	acatatacaa ataaaatgaa taaaactaaa aaaaaaaaaa gggcgg
CD677602.1	Eye trabecular meshwork	tgacatatac aaataaaatg aataaaacta aaaaaaaaaa aaaaaaaaag
BU608452.1	Lung epithelial cells	gacatataca aataaaatga ataaaactaa aaaaaaaaaa aaaaaaaaaa
BU070239.1	Insulinoma	tttttttttt tttttagttt tattcatttt atttgtatat gtcaaaatac atttttattt
BQ272332.1	Insulinoma	tttttttttt tttttagttt tattcatttt atttgtatat gtcaaaatac atttttattt
BM970383.1	Lung epithelial cells	tttttttttt ttttttttag ttttattcat tttatttgta tatgtcaaaa tacattttta
BM509287.1	Insulinoma	tttttttttt tttttagttt tattcatttt atttgtatat gtcaaaatac
BI492551.1	Fetal cochlea	tttttttttt tttttagttt tattcatttt atttgtatat gtcaaaatac
AW675588.1	Cervix	tttttttaatt tttttagttt tattcatttt atttgtatat gtcaaaatac
AW516268.1*	Endometrial adenocarcinoma	tagttttatt cattttattt gtatatgtca aaatacattt ttatttccaa

AW452563.1	B cells	tttttttttt tttttttagt tttattcatt ttatttgtat atgtcaaaat acatttttat
AW021595.1	Fetal cochlea	acatatacaa ataaaatgaa taaaactaaa aaaaaaaaaa aaa
AI439179.1*	Lymphoma	tagttttatt cattttattt gtatatgtca aaatacattt ttatttccaa
AI453664.1*	Stomach adenocarcinoma	tagttttatt cattttattt gtatatgtca aaatacattt ttatttccaa
AA830346.1	B-cell	tttttttttt tttagtttta ttcattttat ttgtatatgt caaaatacat
AI281137.1*	Colon adenocarcinoma	agttttattc attttatttg tatatgtcaa aatacatttt tatttccaaa
AI281011.1*	Colon adenocarcinoma	agttttattc attttatttg tatatgtcaa aatacatttt tatttccaaa
AA262664.1*	B-cell	agttttattc attttatttg tatatgtcaa aatacatttt tatttccaaa
AW968416.1§	Colon carcinoma	gttttattca ttttatttgt atatgtcaaa atacattttt atttccaaaa
AW967905.1§	Colon carcinoma	gttttattca ttttatttgt atatgtcaaa atacattttt atttccaaaa
AU310314.1§	Neuroblastomna	ttagttttat tcattttatt tgtatatgtc aaaatacatt tttatttcca
CK300902.1	Retina	tttttttttt ttttttttag ttttattcat tttatttgta tatgtcaaaa
BQ189797.1	Fetal eye	gacatataca aataaaatga ataaaactaa aaaaaaaaaa aaaaaaggac
BE645430.1	Prostate	ttttagtttt attcatttta tttgtatatg tcaaaataca tttttatttc
AW316726.1*	Meningioma	tagttttatt cattttattt gtatatgtca aaatacattt ttatttccaa
AW264811.1*	Lung squamous cell carcinoma	tttaggttta ttcattttat ttgtatatgt caaaatacat ttttatttcc
AI678890.1	Stomach adenocarcinoma	tttttttttt tttttagttt tattcatttt atttggatat gtcaaaatac
AI082593.1§	Pooled	tagttttatt cattttattt gtatatgtca aaatacattt ttatttccaa
BM511639.1§	Insulinoma	tttattcatt ttatttgtat atgtcaaaat acatttttat ttccaaaata
DB507086.2	Testis	taaaaatgta ttttgacata tacaaataaa acgaataaaa ctaaaaaaac g
CK300002.1	Retina	tttttttttt agttttattc attttatttg aatatgtcaa aataaatttt
AI923578.1*	Endometrial adenocarcinoma	tagttttatt cattttattt gtatatgtca aaatacattt ttatttccaa
AI342464.1*	Uterus	tattcatttt atttgtatat gtcaaaatac atttttattt ccaaaatagt
AI926946.1*	Prostate	agttttattc attttatttg tatatgtcaa aatacatttt gtatttccaa
AI817061.1	Lung squamous adenocarcinoma	ttttagtttt attcatttta tttgtatatg tcaaaataca tttttatttc
BQ186919.1	Eye	aatttatttt gacatataca aataaaatga ataaaactaa aaaaaaaaaa atataaggac
BE965957.2	Leiomyosarcoma	tttttttttt tttttttatag ttttattcat tttattggta tatgtcaaaa
AA282476.1*	B-cells	agttttattc attttatttg tatatgtcaa aatacatttt tatttccaaa
AI971777.1	Fibrothecoma	tttttttttt tagttttatt cattttattt gtatatgtca aaatacattt
AI284479.1*	Esophagus squamous cell carcinoma	aaaatagttt tattcatttt atttgtatat gtcaaaatac atttttattt
H89328.1	Cochlea	Taaaaatgta ttttgacata tacaaataaa atggaataaa actaaaaaaa aa

AW149875.1*	Endometrial adenocarcinoma	tagttttatt cattttattt gtatatgtca aaatacattt ttatttccaa
H89329.1*	Cochlea	cattttattt gtatatgtca aaatacattt ttatttccaa aatagtgggt
DB345733.1§	Thymus	cacatttaaa atagttttat tcattttatt tgtatatgtc aaaatacatt
AW131950.1*	Kidney tumour	ttagttttat tcattttatt tgtatatgtc aaaatacatt tttatttcca
AI868665.1§	Pooled	tattcatttt atttgtatat gtcaaaatac atttttattt ccaaaatagt
BI492771.1*	Fetal cochlea	ttatttgtat atgtcaaaat acatccttat ttccaaaata gtgggttttg
Z40397.1*	Brain	attcatttta tttgtatatg tcaaaataca tttttatttc caaaatagtg
AI472433.1*	Colon adenocarcinoma	aaaatagttt tattcatttt atttgtatat gtcaaaatac atttttattt
N057465.1*	Breast carcinoma	ataaaaatgt attttgacat atacaaataa aatgaataaa actattttaa
FN057465.1*	Breast carcinoma	ataaaaatgt attttgacat atacaaataa aatgaataaa actattttaa
BU960397.1	Pool	aaaatgtatt ttgacatata caaataaaat gaataaaact aaaaaaaaaa
BG169053.1	Hypernephroma	ataaaaatgt attttgacat atacaaataa aatgaataaa actaaaaaaa
AA903120.1	Leiomyosarcoma	tttttttttt tcacacattt aaaatagttt tattcatttt atttgtatat
BI966891.1	Pancreas	ttttttttt catggcaaaa tacattttta tttccaaaat agtgggtttt
DW461699.1	Liver	tacaaataaa atgaataaaa ctattttaaa tgcgcaaaaa aaaaaaaaaa

Table S5. Collection of EST clones supporting terminator region T2 approximately 2360 bp downstream of the stop codon. Accession numbers are hyperlinked to the respective GenBank submission. Sequences at the end of each clone were taken directly from the GenBank submission, note that most are in antisense direction. Cleavage sites are highlighted in turquoise unless reliable identification was not possible. *Clones without a clear polyA stretch but with annotation that an oligo dT was used in the cDNA synthesis. §Clones without a clear polyA stretch and no annotation about an oligo dT being used in the cDNA synthesis.

Accession number	Tissue or cell type	Sequence at end of clone
AI061415.1	Gessler Wilms tumour	tttttttttt ttttttcaga ttctgcatac agacagcttt atgtaaaata
DB524753.2	Testis	atgtttatat attttacata aagctgtctg tatgcagaaa aaaaaag
BM968507.1	Lung epithelial cells	tttttttttt ttttttctgc atacagacag ctttatgtaa aatatataaa
AW673535.1	Cervix	ttatatattt tacataaagc tgtctgtatg cagaaaaaaa aaaaaaag
AW273092.1	Colon carcinoma	tttttttctg catacagaca gctttatgta aaatatataa acatttgtcc
AA913505.1	Pooled	tttttttttt tttctgcata cagacagctt tatgtaaaat atataaacat
W32762.1	Parathyroid tumour	nttttctgca tacagacagc tttatgtaaa atatataaac atttgtccaa
AA047292.1	Uterus	nttttttttt tttctgcata cagacagctt tatgtaaaat atataaacat
DB356402.1 §	10-week embryo	ctgcatacag acagctttat gtaaaatata taaacatttg tccaaattgg
BE328265.1	Wilms tumours	tttgcataca gacagcttta tgtaaaatat ataaacattt gtccaaattg
AW731690.1 *	Cervix	tgcatacaga cagctttatg taaaatatat aaacatttgt ccaaattggt
AI004347.1	Fetus	tttttttttt tttttttttt ttctgcatac agacagcttt atgtaaaata
T89964.1 *	Lung	tgcatacaga cagctttatg taaaatatat aaacatttgt ccaaattggt
AA132555.1 *	Colon carcinoma cell line	gcatacagac agctttatgt aaaatatata aacatttgtc caaattggta
AI890988.1	Endometrial adenocarcinoma	tttttttttt tttttttttt ttctgcatac agacagcttt atgtaaaata
AI004376.1	Testis	ttttgcatac agacagcttt atgtaaaata tataaacatt tgtccaaatt
AA027817.1	Uterus	tttttttttt ttttnctgca nacagacagc tttatgtaaa atatataaac
DB373613.1 §	Placenta	ctgcatacag acagctttat gtnaaatata taaacatttg tccaaattgg
AW799002.1 §	Uterus	aaaacagctg atgtaaatat ataaacattg tccaatggta cacagatgca
DN914761.1 §	Breast cancer cell line	cggaaacaga cagctttatg taaaatatat aaacatttgt ccaaattggt

Supplemental Figures

Exon 1$^-$ (primer E1$^-$f)

```
     1 gaggcAGATCTGCCCTGTAGAGCCTTGCGGTTCCACTGCTGGCCTCCGGATTCCCGGGAGCCCCCAGCCCGACAGGACAACCTTCCTTCCCCGCTTCCTCCTTTCCAGgtctgtcccagc    120
   121 ccccgctgcctgggccacacccgcttccctctggtggcccagccaccaccgccgcagttcgcgggagcgctcttcctggggcgggcacaggcccagggcgcatgcgcactggtcgaaggg    240
```

Exon 1 (primer E1f, E1′f, E1r)

```
   241 cgcctcccagtccggatctcgcgaagttacgttagattggccgccggtgacgggtggccgtgctgggggcgggagggcttctgtggtaggaagggaggtccgctcggccgggtgcgccgc    360
   361 cccagtgctctgtgggatactggaagtctcgagcgtcggctccgggttcccagccctcctctggcccgcactcatagaaacattcacacacccccctgcctcccctctcttccctctccgc    480
   481 ctccccccttccccccctcgcgataagaagACCCGGCGGCAGGAGAGGGGATGAAGATGGCGGACGCGAAGCAGAAGCGGAACGAGCAGCTGAAACGCTGGATCGGCTCCGAGACGGACCT    600
   601 CGAGCCTCCGGTGGTGAAGCGCCAGAAGACCAAGGTGAAGTTCGACGATGGCGCCGTCTTCCTGGCTGCTTGCTCCAGCGGCGACACGGACGAGGTCCTCAAGCTGCTGCACCGCGGCGC    720
   721 CGACATCAATTACGCCAATGTGGACGGACTCACTGCCCTGCACCAGgtctgtgcgccccgccgccggctctcctgaccgtgagggttctcgtcggcctcctgctcgggcctgccctcagt    840
```

Exon 1$^+$ (primer E1$^+$f, I1r)

```
 21481 gctgaattctcatatccacttctgcatctagcctattgtgatgtcacatacaagctcctggaaaactccATCATATGCTTGTAAGAGAATGAGAGTATTAACCTTGTAGACTTCTGTAAG  21600
 21601 GGGCTAAGAGAAACACTGAGCTAAATAATTGAGGGGATATACTATGTTCATGGCTTGAAAGACTGTAAgtattgtaaagatgttcttccgaaaccaatcacagatttaatacaagtccaa  21720
 21721 acaaaatcccagatatgtgtgcattgtctgtttgtgtatgcatgtgtgaaaaatggtaaattgatataaagatgaaaaagaccaggcacagccaagtcaatctttaggaacaagaaagtg  21840
```

Exon 2 (alternative ATG when transcription starts at E1+)(primer E2r)

```
 62521 agGCTTGCATTGATGACAATGTTGATATGGTGAAGTTTCTGGTAGAAAATGGAGCAAATATTAATCAACCTGATAATGAAGGCTGGATACCACTACATGCAGCAGCTTCCTGTGGATATC  62640
 62641 TTGATATTGCAGAgtaagccagttctgtgttttcattttattctaatactgtttgacagacctgaatgtttattgaagtgataaagataaatgagtgaactcttatattactttaattca  62760
```

Exon 2$^+$

```
 86521 gtcacacagGTGTCAACAGGCAGAGGTGGGAACTGAACCCGAAGTCTGCAGAGCATAAAAGTTTCTGCTTTTTTCACCCCTCCAACCTTTGAATTGCTTTTGGAGACTATTTATGGTAGA  86640
 86641 TAAGAATTAATCCACCATAGGATTAATCAGTCCgtgagattttgtttattattatttattattatttttttcaaatgtgcctttgtaacatttacatttgcaagtaatagttctagagtgc  86760
```

Exon 3

```
 90001 taatatcttttgcttaactatttttcctttggttttacagGTTTTTGATTGGTCAAGGAGCACATGTAGGGGCTGTCAACAGTGAAGGAGATACACCTTTAGATATTGCGGAGGAGGAGG  90120
 90121 CAATGGAAGAGCTACTTCAAAATGAAGTTAATCGGCAAGgtaaaataaaaagcaacctaaatggaaaaatgttttagaaagaatgtgatacttttccagcaaaaatatttgttgacattg  90240
```

Exon 4 (primer E4f, E4′f)

```
102961 ttttatcagGGGTTGATATAGAAGCAGCTCGAAAGGAAGAAGAACGGATCATGCTTAGAGATGCCAGGCAGTGGCTAAATAGTGGTCATATAAATGATGTCCGGCATGCAAAATCTGGAG 103080
103081 GTACAGCACTTCACGTTGCAGCTGCTAAAGGCTATACGGAAGTTTTAAAgtaagtattgtttttttacagagtttttctattttagtttgtcttctttattcattgttccttttcttaaaa 103200
```

Exon 5

```
106921 catgatagatagcttttgatcatgatcatttatttctagattttaatgattgtctaattcactgtaatttctttagACTTTTAATACAGGCAGGCTATGATGTTAATATTAAAGACTATG 107040
107041 ATGGCTGGACACCTCTTCATGCTGCAGCTCATTGGGGTAAAGAAGAAGCATGTCGAATTTTAGTGGACAATCTGTGTGATATGGAGATGGTCAACAAAGTGgtaggcgttttaaacgtat 107160
```

Exon 6

```
113161 tgtaaaaaatgttatccaacgtctttatataaaattatacagaattactgaaggcattttatgatttattgacttttgaccatcaccatccccccttgtttcctacgtagGGCCAAACAG 113280
113281 CCTTTGATGTAGCAGATGAAGACATTTTAGGATATTTAGAAGAGTTGCAAAAGAAACAAAATCTGgtatgtttgatgactaataattgcctatattacttaccttaataattttgaattt 113400
```

Exon 7

```
114241 agtaatagtatcttaatagttttcttattttcctttgcataactttgtacaaagCTCCATAGTGAAAAACGGGACAAGAAATCTCCACTAATTGAATCAACAGCAAATATGGACAATAAT 114360
114361 CAGTCACAGAAGACCTTTAAAAAgtgagtaaaatattcaagtacatttgttatttttgttaatgagttggcatagtggccacaggcatgattattgtggatctgggaagaatgaatatat 114480
```

Exon 8 (primer E8f,E8r)

```
114481 tttaaccttttgtatttctaatcatttgaacactgttttcctttaacagCAAAGAGACGTTGATTATTGAACCAGAGAAAAATGCATCCCGTATTGAATCTCTGGAACAAGAAAAGGTTG 114600
114601 ATGAAGAAGAAGAAGGAAAGAAGGATGAGTCTAGCTGCTCTAGTGAAGAAGATGAGGAAGATGACTCGGAATCAGAAGCTGAAACAGgtaaaattaaaagataaatgcattttcgttgaa 114720
```

Exon 9

```
117841 taacaaaaccttaaacaaaaatggaatataaaatgatttttcccagccattgagaccaaatatatatggaatcttgactcttaattgtttgtctttaatagATAAGACAAAACCCCTGG 117960
117961 CTTCTGTAACTAATGCCAACACTTCTAGTACACAAGCAGCTCCTGTAGCTGTTACAACACCTACTGTGTCATCAGGTCAAGCAACACCTACATCACCTATTAAAAAGgtaagccaaaatt 118080
```

Exon 9$^+$ (primer I9f, E9$^+$r)

```
124441 tgcatttttagggttgttggaaagcccttaaaatagcataaatatttgtacataaaattagttttttattatagttgaaatagttaatgtttggtggggaaatcagtaaatttaaggctgt 124560
124561 gatgttaaaaaaaaaagtattataattggaaaaatatcagttactagttttttgttgattatattggacacaaaattttttaaccagagtatatacatggattaaaaacaaaattcatgtg 124680
124681 cagtagtttgttcagtcacatgctatattatctactctgtatgccttttaattgctttagaagtttattagattttttttcagattatctcatgctaaaattagaacttgttcccagtgac 124800
124801 ttttaaagattggatcattacccaattattggggttaattataaaattattttcaaatgaagaaaatattttttgttcataacttcccttttctgttttcatgctttcaatctttgtctc 124920
124921 tttacctgtcttattttaattggctttagTATGATTTCATTGCTCCTATCATGCCTGTGGTGGAATCAGTAGATCCTGCATCATGGAGGCAGGGCTTGCGCAAAACTGGCATTGTTCTTG 125040
125041 TGCCCAGTAAGGGTGAAAAATCTATGgtgaggcatttctatgtgcaagaatttatattaggttacaacataacaagtttaatataaattggttccaaagatgcatggttttaagtcctta 125160
```

Exon 10 (primer E10r)

```
125401 tcagctttacaaattctttttttcttttcctaaaaaataaaataaacacagTTTCCAACCACAGCTACAAAAAATTTCTCCCAAAGAAGAAGAGAGAAAAGATGAGTCTCCTGCAACTTGGA 125520
125521 GGTTAGGACTTAGAAAGACGGGCAGCTATGGTGCACTTGCTGAAATCACAGCATCTAAAGAGGGTCAGAAAGAAAAAGATACTGCAGGTGTTACACGTTCAGCTTCAAGTCCCAGACTTT 125640
125641 CCTCCTCTTTGGATAATAAAGAAAAGgtaatctatttcacagtctggtttctatgaaaactgttatgatttcttttccaggaacatcaaatctggtagattttctgctagttatctttag 125760
```

Exon 11 (primer E11f)

```
126841 gtacccaaattaattttttacttattttttttccctgttgtgtagGAGAAAGATAGTAAAGGAACTAGGCTTGCATATGTTGCACCTACAATACCAAGACGACTAGCCAGTACATCTGACAT 126960
126961 TGAAGAGAAAGAAAACAGgtgagtttatttttgatttgctttatgcattcactataaaatctttattatctagcattagagttaatacatgagcattgtaattatgtgcggatatgtattg 127080
```

Exon 12 (primer E12f)

```
128041 ttcagcttatactaatatttactagtgtaccgctttcttaatcctattttaaaattatgtaccagaatctgaagggtgttcttttaacagAGATTCTTCAAGTTTGCGAACAAGTAGTTC 12816
128161 ATATACAAGGAGAAAATGGGAAGATGATCTTAAAAAAAAATAGCTCAGTTAATGAAGGATCAACGTATCATAAAAGgtaataatactattcttaagcggtttttatgtattcatttataaa 128280
```

Exon 13

```
129121 tgtttagTTGCTCCTTTGGTAGAAGACAAGATGATTTGATTAGTTCTAGTGTTCCAAGCACCACATCAACACCAACAGTTACCTCTGCAGCTGGGCTTCAGAAAAGCCTGCTTTCCAGCA 129240
129241 CAAGCACTACTACAAAGATTACAACGGGTTCTTCCTCAGCAGGCACACAAAGCAGgtaagtcacagatacattttgtgctcaggtctactgtgtgcttatgtatgtgcatgcgtttttgt 129360
```

Exon 13b (alternative splicing acceptor site)

```
129121 tgtttagttgctcctttggtagaagacaagatgatttgattagTTCTAGTGTTCCAAGCACCACATCAACACCAACAGTTACCTCTGCAGCTGGGCTTCAGAAAAGCCTGCTTTCCAGCA 129240
129241 CAAGCACTACTACAAAGATTACAACGGGTTCTTCCTCAGCAGGCACACAAAGCAGgtaagtcacagatacattttgtgctcaggtctactgtgtgcttatgtatgtgcatgcgtttttgt 129360
```

Exon 14 (primer E14r)

```
129601 tgtagtcatgtgttttcagctgctagaaagtgctttcattgtttagctgttttcctttttctctttttttgtgttgctgcttatgatgctatagTACCTCAAATCGTTTGTGGGCTGAGGAT 129720
129721 AGTACTGAGAAAGAAAAGGACAGTGTTCCTACGGCAGTGACCATTCCTGTTGCTCCAACTGTTGTAAATGCTGCAGCTTCTACCACAACCCTGACTACAACTACTGCTGGCACTGTCTCC 129840
129841 TCCACAACAGAGGTCAGGGAGAGACGCAGgtgtgtaaaggtcataacagtactgctgatatatcttgcccagtgatgaaagttgtttttttgttttaaaaaagatcttgtgaggttgatgg 129960
```

Exon 15
```
136801 ttcatatgcattgatagatatttacattcacaggtattgtatttacaactaacgatttttcaatttactactgtagATCATACCTCACTCCTGTTAGGGATGAAGAGTCTGAATCCCAAA 136920
136921 GAAAAGCAAGATCTAGACAAGCAAGACAATCTAGAAGATCAACACAGgtatagttaatgtaattaatatataaaacttacataagtagcatataaaatatatatacacacactgacataa 137040
```

Exon 16 (primer E16r)
```
138001 taaaatattccttgaaacaagctttaatgcatctcataattatattgatctttttgtgtgtgcagGGAGTGACATTAACTGATCTTCAAGAAGCTGAGAAAACAATAGGAAGAAGTCGTT 138120
138121 CTACCCGAACCAGAGAACAAGAAAATGAAGAAAAAGAAAAAGAGGAAAAAGAGAAACAAGATAAAGAGAAACAAGAAGAAAAGAAGGAGTCAGAAACATCTAGAGAAGATGAATATAAAC 138240
138241 AAAAGTACTCCAGAACGTATGATGAGgtaacagtatatcaaaagcacatttaagtcaaatggtatgaatagtccttatgatccttaacatacaagagatttatataccaacaaaccatat 138360
```

Exon 17
```
138481 caaatgggttttgatcttaatgtttcacagACTTACCAGCGTTATAGGCCAGTATCAACTTCAAGTTCAACCACTCCATCCTCTTCACTTTCTACTATGAGCAGTTCACTGTATGCTTCA 138600
138601 AGTCAACTAAACAGGCCAAATAGTCTTGTAGGCATAACTTCTGCTTACTCCAGAGGAATAACAAAAGAAAATGAAAGAGgtaagaataccaaattttgaaaacactttgctttcttcata 138720
```

Exon 18
```
139681 gttcattgcaatccttaacagAGGGAGAAAAAAGAGAAGAGGAGAAAGAAGGAGAAGATAAATCACAACCTAAATCAATCAGAGAACGACGACGACCAAGAGAGAAAAGAAGATCTACAG 139800
139801 GAGTTTCATTTTGGACACAAGATgtgagaacctattaaaatacattatttgatagtattcttgatagtattcttaatgtgtccaaaaatacatactaataatttcttatatgccttagat 139920
```

Exon 19 (primer E19f)
```
141481 ttgaaattattactaaaatttcctaaaatataaattggcaatctgtttttagAGTGATGAAAATGAACAAGAACAACAATCAGACACAGAAGAGGGATCCAATAAGAAAGAAACTCAGgt 141600
```

Exon 20
```
144961 tttctctttctttccagACGGATTCCATTTCTAGgtatgttttatttatttttaactgacatttttttcttatttttatctttgttaaaaatgtgttgctaggaatttatggattttgt 145080
```

Exon 20⁺
```
145681 aagtgtcacctaattcaaaattatccatcttttcagagaacctccactatatccttgattttgatctccattccatttctcttatcattgttaaccacagCCCTGTAAGACCAGTAATAA 145800
145801 ATTTCTGTCTACTGATGATTCTATTGAATTGAAATGCAGAGCCAATGGCATCTTTCAGAAAAGgtaacctgaatttcagaaccagataataagagaattctcctttaccagtaagatatg 145920
```

Exon 21 (primer E21f)
```
146641 tataacaaggtttttatttaaattaatcttttgaagATATGAAACCAGTTCTACATCAGCTGGTGATCGATATGATTCCTTGCTGGGTCGCTCTGGATCATACAGTTACTTAGAAGAAA 146760
146761 GAAAACCTTACAGCAGCAGGCTAGAAAAGGATGACTCAACTGACTTTAAAAAGgtacttgggttatttagttatatttaataaaaagttatcttttatgttgagatactcattcaaatat 146880
```

Exon 22
```
148921 agaaattttaaagtaataggagagcagaaaagtaatttgttcttttaccctaatttttatagCTTTATGAACAAATTCTAGCTGAAAATGAAAAGCTGAAGGCACAGCTACATGATACAA 149040
149041 ATATGGAACTAACAGATCTTAAATTACAGTTGGAAAAGGCCACCCAGgtttgtacccttttctttttgttactctcaaggtaatggttgtgatttatcccatctaaaaatggtattaaaa 149160
```

Exon 22⁺
```
152761 gtttgtacagagatcaatgtaaataaaatctaagatgccactgtttttttcttttttttttcttttttttgttttgttcttaaactgtgcctgtgcttaatgtccagAGCTCTCCAGTACAG 152880
152881 GATACAACAGgtaggctaaaacccaaggtgcttttccatcccttaaagccaagcttatatatcaggatttcatcagctttgagctggtcagatagtacaggaagtggaaaaaatgaaagg 153000
```

Exon 23
```
153481 aacctgtgttttactccacttgaatattgtgatttaatacctattaaaagagaattactgtggtcacccctgaactttttttgcacttatctcttttataatttcttactttgttgcagAG 153600
153601 ACAAGAAAGATTTGCTGATAGATCACTGTTGGAAATGGAAAAAAGGgtaagtgtcccacttattaaataattattttttttctttcttgttttaggtaaacaatggtaggtttttgtttt 153720
```

Exon 24
```
156001 aaatgtactgcttgactaaatatcagcattatttattaatctgggaaagagtgtcttggaaatagtttcagtgtgattaaactaaatttaaatggtttgtatctgaaagGTGACCGGCAA 156120
156121 GAGTCAGTATCTTCTGGGCGgtatgacctaaccagttttacagctcatcaattactgctgcttgctttttgcataacaccccagaatgcttatcatttaagggagaataaaacagtacac 156240
```

Exon 24b (alternative splicing donor site)
```
156001 aaatgtactgcttgactaaatatcagcattatttattaatctgggaaagagtgtcttggaaatagtttcagtgtgattaaactaaatttaaatggtttgtatctgaaagGTGACCGGCAA 156120
156121 GAgtcagtatcttctgggcggtatgacctaaccagttttacagctcatcaattactgctgcttgctttttgcataacaccccagaatgcttatcatttaagggagaataaaacagtacac 156240
```

Exon 25
```
156841 ggtaccttaaatcgatctagGAACGAAGAGCTCTAGAAAGAAGAATATCTGAAATGGAAGAAGAGCTCAAAgtaagtaaactatgttacttatgagagagagtgctgtttatgtattaat 156960
```

Exon 25 (alternative stop TGA when E24 or E24b spliced in)
```
156841 ggtaccttaaatcgatctagGAACGAAGAGCTCTAGAAAGAAGAATATCTGAAATGGAAGAAGAGCTCAAAgtaagtaaactatgttacttatgagagagagtgctgtttatgtattaat 156960
```

Exon 26 (primer E26r, E26′r)
```
159361 ggtgaaaatataacatttattaagaataatgttttatcttttaatattgaaggtaaaacttgatcttcaattttctctttatagATGTTACCAGACCTAAAAGCAGACAACCAGAGGCT 159480
159481 AAAGGATGAAAATGGGGCCTTGATCAGAGTTATAAGCAAACTTTCCAAATAAAAAAAAAAAAAGCAGCAAGTAATGGAATTGCACATATTAGTAACCCAGTGGACCATAATTGGCAGTCA 159600
159601 CTGGAAGTCTGGGAAGAATCCTTGGAGACTGTCATTTTCGGATATCCTGCCAAATGCCCTCTTATCTAGAATTTTTGTTTCATTTTGTTTAATTTTCTGGGGTGTTTTTGTTGTTGTTGG 159720
159721 TTTGTTTTTTGTTTTTTTTTTTTAATCAAGACCATTGTTTCATGTTAATGCAGCTGCTGAGAAGATTTTTTTTTAATGACTGAGAAAACTTGTTTACAGCTCCAGCATATAAGGAAAGTG 159840
159841 TTCAAGGCCAGATATGCCTCAGATATTTAACCAGTAAGCCTTAGTTGTACATAAATACTTTTGTGTCAACAAAAACTTTCAGCTCTCACAGAAGACAGTTACTCAACATTTTTTGATGTG 159960
159961 CCACAGTTTCGAGTTTTTCGATATTTAAATTTTTTGGCTTTTCATCTAAGTTTGGGTTTGTATTTTTTCCTTCTAAACTCTTCATGTGGCAGAGTCTTCTATGTTTTCACAGCTTTTTCA 160080
160081 TTACAGAAAAGAACACTTGCTCTTCTGTGATTATTGTCATGTATTAGGCTAATGCTGTGTTGTCTCCCACCTGGAACTGAATTGCTTGGTGGAACATATGCTTTCACTGTTTGTGCAATA 160200
160201 TGCATTTATTTCTTATATGAATGCTTTAAAGTCATTTGAGGTTAGATCTTTTAATTCCTATTTTCTGCTTCATTGGTCACTTTTTTTTTTATTGTAGTATAAGATGTTAGATTCTGTAATC 160320
160321 TTCACATTCATTTTAGCAGGTACTGAGTGATGCTGTATATACAAATAAGTGTATTGTTTTGATTTTTAGACCACCACATGGCATGCTTGACTATTTCTTATTTCAAATGTCTGCTAATGC 160440
160441 AGAGTAGGCTACTCCATGATAGTGTTAAAAAACAAAATTTGCTAACAATGTGATATAAAGACTTTAAAAGTTACACATTATGTGGAGCCCTATCTTTACAAAAGTTTCCTACTGTAAAGT 160560
160561 GCTTTTATTTTCAGTTTTCACTTGATAGTACTCAACCATAATTAAAGTTGCATAAGATAATTGCTTTACATTTCACATACCTATATTTATCTGAGTGCTGTCTAAAACTGTTGTGCTAGC 160680
160681 CAAAGTAATGCTATGAAATCATTTGCAGAATTAACCCGTGAGTTAATGTTAAATGCACTGTTATTGCCATGTGAAGAGGCATCGACTTTGATACCACCATCATGTTCAGACCATTTTATA 160800
160801 CATTTCAGTGGCCTTTTTTTTTTTTTAAGGAAAAAAAAAGCGCAAAACCAAGTACATAGTGACGATGGCTTTTATTTGGACAAATAGCTTTTATATTTTCATTAAACCATGCAAAAAATAC 160920
160921 TACATCTTTCTGGCACATAACTGTCTCCTTAACCACTGAACAGTTCAGCCATTTGAATAAATTGTACATTGTAAAGCTTATAGTAGCTGATTGTATTATTGATTGTATTGTATACTATAT 161040
161041 TAAATGTGAATTTGTACCCCTTCATTTTAAAAGGTCATTGTGTTCTACTGCCTATTTTAGATTACTTTGGGGTTGGGGAAGGGTGTTTTGGTAAGCAGGATTTTAAGTCCTCTCTCTCTC 161160
161161 GATTGGTTTTATGAAGATATAAGGTTATGCTCTTACTACCAAGCTCAGGATTCTTGATTTTAAAGGTACAAGGATAGTTGTGGGAGTTTTATTTTTGGTTATATTTGAACTGTATGAATG 161280
161281 GTAGGTCTGAATATTGAGAATTTCCATGGTCTTATGTGGAGGTAAAATATACAGATAACTGATCAGTTTGAGTGACTGCAACATGTTCTGGTTGCACAACAGTTAGGCATGCTATGGTTT 161400
161401 ATATTTGTGAAGTTTGGATGCCTGTGAAGAATGATTAAATACATGTTTCTAATATTTTAGTGTTTTGTATTTAGGTTATTTATTCTTTGTATCTAAAACTGAACAGCTACTGTGCTATAT 161520
161521 TGATTTTATTGGTAGTATTGAGCAGACCTTGTTTCTGCATGAAACTAGTTGCAAAACCCACTATTTTGGAAATAAAAATGTATTTTGACATATACAAATAAAATGAATAAAACTATTTTA 161640
161641 AATGTGTGAACTTTTAATGAAGACATTTTAAATTTTGTTCTGAGATATTTAAATTCTGCATGGGTATTTTTTCACTAATTGCTTTACTTGGATACCAATTTGAATAATTTCTGAAATTGT 161760
161761 AATATTGTGATAATTTGCACAGCATTGTACATGCACATCTGTAAATGCAATCTTAATTGCCATATTTATGCAATCTGTGTACCAATTTGGACAAATGTTTATATATTTTACATAAAGCTG 161880
161881 TCTGTATGCAGAATCTGA                                                                                                      161898
```

Exon 26 (when E24 or E24b spliced in) (primer E26r, E26′r)
```
159361 ggtgaaaatataacatttattaagaataatgttttatcttttaatattgaaggtaaaacttgatcttcaattttctctttatagATGTTACCAGACCTAAAAGCAGACAACCAGAGGCT 159480
159481 AAAGGATGAAAATGGGGCCTTGATCAGAGTTATAAGCAAACTTTCCAAATAAAAAAAAAAAAAAGCAGCAAGTAATGGAATTGCACATATTAGTAACCCAGTGGACCATAATTGGCAGTCA 159600
159601 CTGGAAGTCTGGGAAGAATCCTTGGAGACTGTCATTTTCGGATATCCTGCCAAATGCCCTCTT... etc
```

Figure S1. Exon composition of *PPP1R12A* and location of PCR primers used in this study. Nucleotide numbering is as per Ensemble transcript PPP1R12A-202. Exons are in upper case except for the part of E1 that is spliced out when transcription starts at E1⁻. Introns are in lower case. Coding sequences are highlighted in turquoise except when E24 is spliced in, in which case the sequence after the alternative stop codon in E25 and E26 is non-coding. Non-coding sequences are highlighted in yellow. Exons of non-sense mediated decay transcripts are highlighted in grey. Translation start and stop codons are highlighted in green and red, respectively. Forward PCR primers are in green characters, reverse PCR primers in red characters except E26’r, that overlaps with E26r and is underlined. Primers spanning exon boundaries are not indicated. Note duplication of E13, E24, E25 and E26 to accommodate alternative splicing variants.

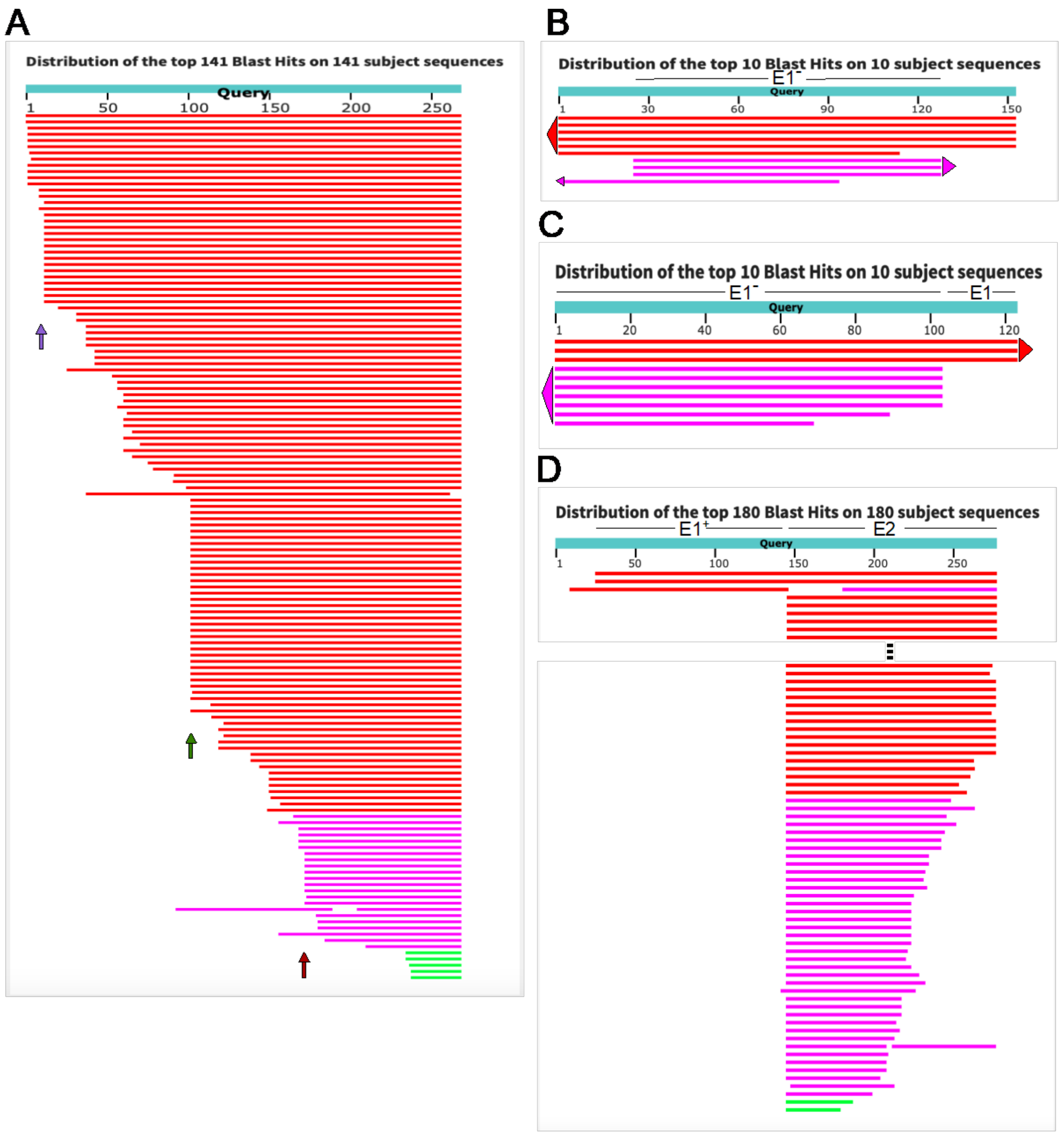

Figure S2. Results of BLASTn searches for ESTs in the transcription start regions of *PPP1R12A*. Horizontal bars represent the extent of the alignment of the database sequences to the query sequence and are color-coded by score: red, pink and green from highest to lowest. (A) EST support for a transcription start in E1. The human EST database was interrogated with the sequences corresponding to the UTR of E1 as per transcript PPP1PR12-208. Arrows indicate predicted transcription start sites and are colored like in Fig. 1C. (B and C) EST support for a transcription start variant at $E1^{-}$. In (B) the human EST database was interrogated with the sequence corresponding to $E1^{-}$ flanked by 25 bases of genomic/intron sequence. The arrowheads indicate the directionality of the clones. Three hits read towards the right and encompass only the $E1^{-}$ sequence. The rest read in the opposite direction and correspond to the overlapping antisense gene *PPP1R12A-AS1*. In (C) the human EST database was interrogated with the sequences corresponding to $E1^{-}$ and 20 bases of E1 as per transcript PPP1R12A-201. The hits are the same as in panel B: the three top ones correspond to EST clones that contain $E1^{-}$ and splice $E1^{-}$ into E1, the rest correspond to *PPP1R12A-AS1*. (D) Support for a transcription start variant at $E1^{+}$. The human EST database was interrogated with the sequences corresponding to $E1^{+}$–E2 flanked by 25 bases of genomic/intron sequence before $E1^{+}$. The output has been truncated (dashed line). The two top hits correspond to EST clones that include $E1^{+}$. One EST that covers only $E1^{+}$ is flanked by intron sequences and may correspond to amplification of contaminating genomic DNA.

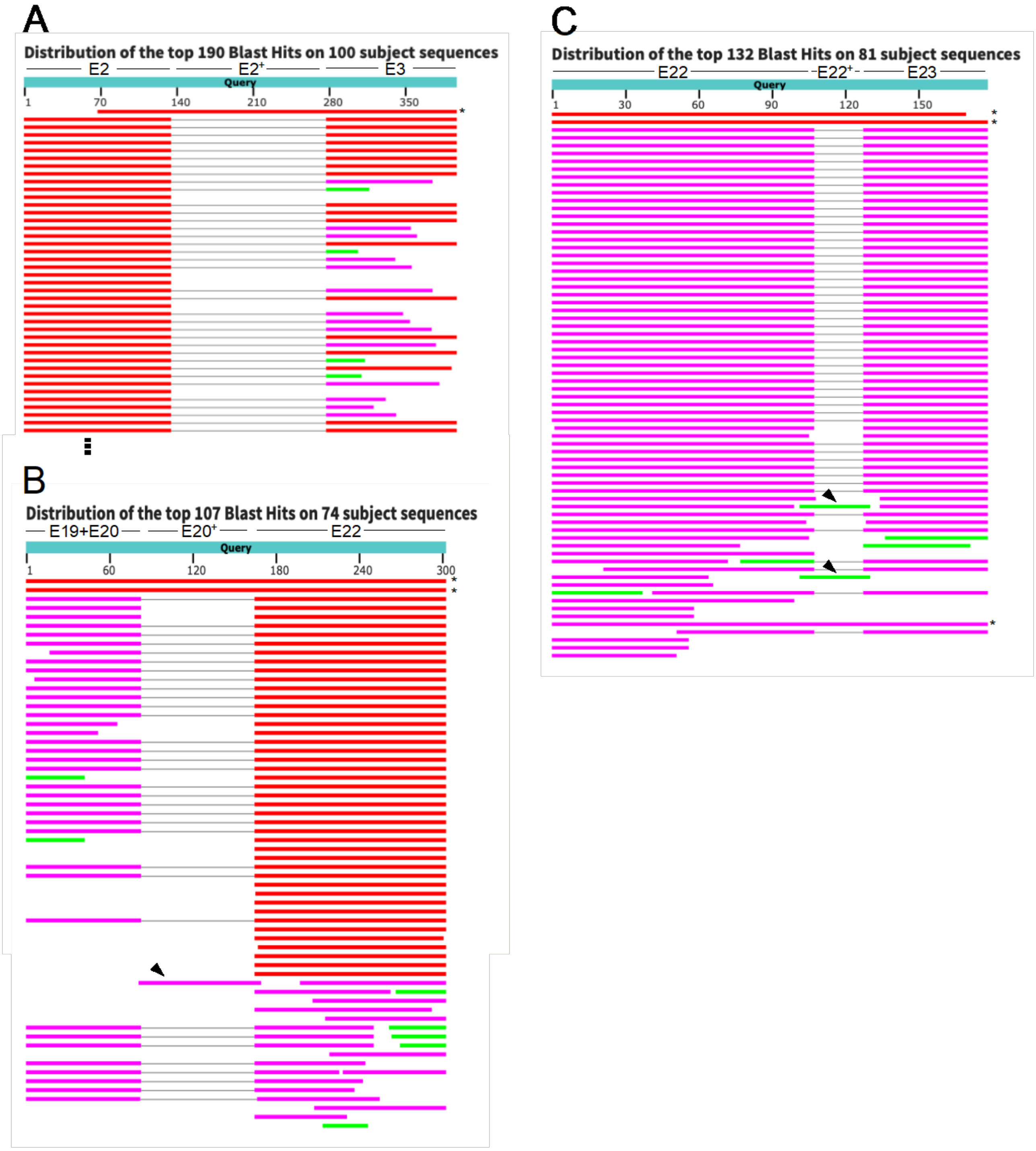

Figure S3. Results of BLASTn searches for ESTs in support of transcripts with non-canonical exons. Horizontal bars represent the extent of the alignment of the database sequences to the query sequence and are color-coded by score: red, pink and green from highest to lowest. Separate aligned regions on the same database sequence are connected by a thin grey line. (A) EST support for a splicing variant with 144 bp exon E2^{+} (transcript PPP1R12A-209). The EST database was interrogated with the sequences corresponding to E2–E2^{+}–E3. The search was limited to 100 hits and only the top 42 hits are shown. A single EST (BP279682.1, asterisk) supports this transcript. The annotation in Ensembl identifies a start codon in E3 that matches the sequence of MYPT1 and interprets the sequences upstream as UTR. It appears more likely that the inclusion of the extra E2^{+} would cause a shift to the canonical reading frame, introducing a premature stop codon, therefore this variant would be a nonsense mediated decay mRNA. (B) EST support for a splicing variant with 83 bp exon E20^{+} (PPP1R12A-217). This exon causes a frame shift and a premature stop. The EST database was

interrogated with the sequences corresponding to E19–E20–E20$^+$–E22. Here E19 was included due to the small size of E20, insufficient for significant alignments. This search revealed two clones that spliced in E20$^+$ (asterisks). One clone that only covers E20$^+$ (arrowhead) contains intron sequences upstream and downstream and may correspond to an intron retain clone. (C) EST support for a splicing variant with 25 bp exon E22$^+$ (PPP1R12A-205). This exon causes a frame shift and a premature stop. The human EST database was interrogated with the sequences corresponding to E22–E22$^+$–E23. This search revealed a single sequence, BG180627.1, containing E22$^+$ (top hit with an asterisk). The second hit appears to correspond to a clone (BF980059.1) with anomalous splicing at the E22$^+$–E23 boundary. One clone that appears to include E22$^+$ (at the bottom, asterisk) corresponds to a misaligned sequence and two more that only cover E22$^+$ (arrowheads) contain intron sequences upstream and downstream and may correspond to intron retain transcripts.

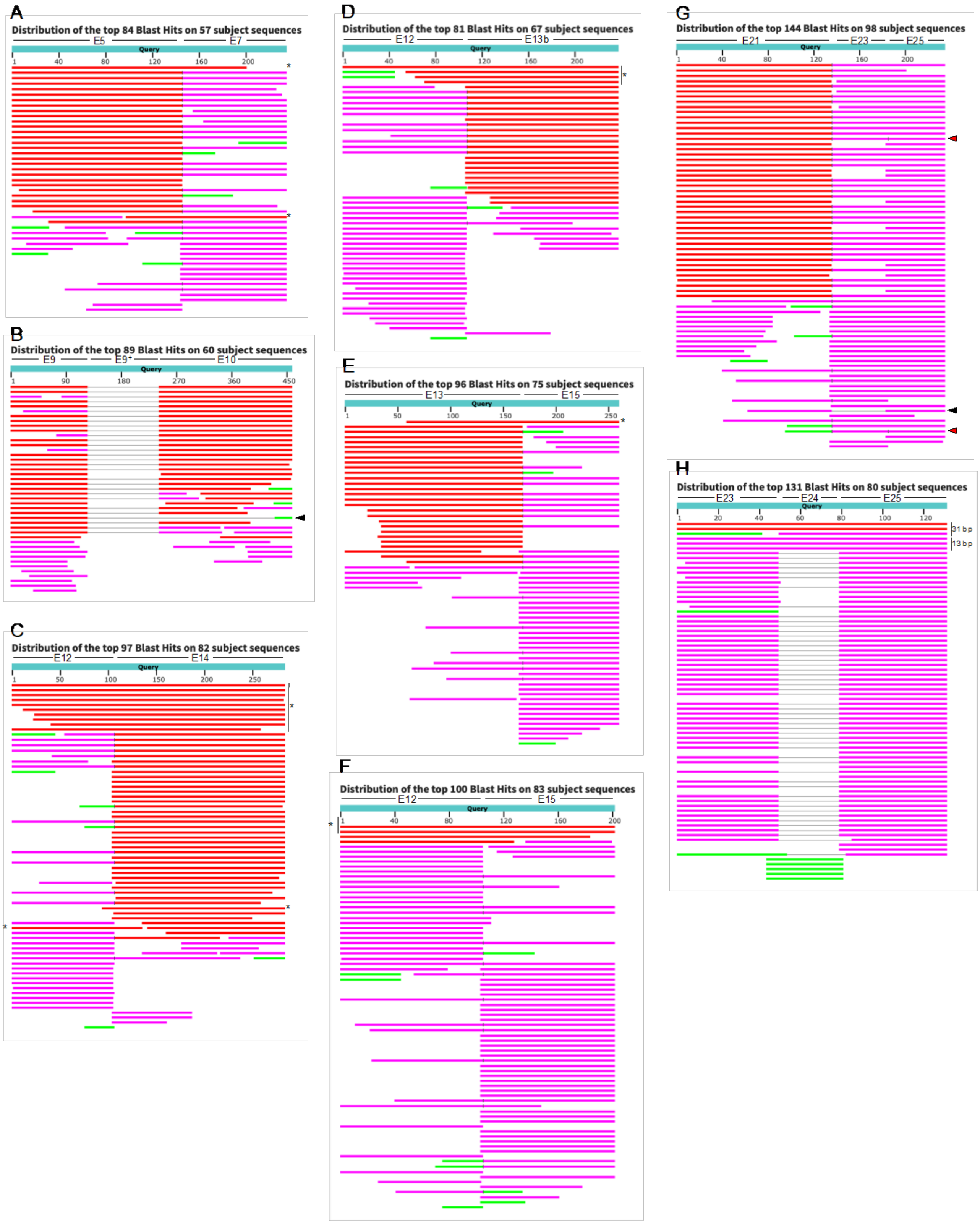

Figure S4. Results of BLASTn searches for ESTs in support of alternatively spliced exons. Separate aligned regions on the same database sequence are connected by a thin grey line. Vertical tick marks indicate intervening sequences not present in the query sequence. (A) Splicing of E6. The EST database was interrogated with the sequences corresponding to E5–E7. The boundary between E5 and E7 is placed at bp 145. Two ESTs were found in which E6 was spliced out (asterisks). (B) Splicing of 117 bp exon $E9^+$ (transcript PPP1R12A-223). The EST database was interrogated with the sequences corresponding to E9–$E9^+$–E10. This search and a further search with the $E9^+$ sequence alone failed to reveal any ESTs including $E9^+$. The sequence marked with an arrow showed anomalous splicing of E10. (C) Splicing of E13. The EST database was interrogated with the

sequences corresponding to E12–E14. The boundary between E12 and E14 is placed at bp 105. Twelve ESTs were found in which E13 was spliced out (asterisks). (D) An alternative splicing acceptor site in E13. The EST database was interrogated with the sequences corresponding to E12 plus E13b, a 32 bp truncated E13. The boundary between E12 and E13b is placed at bp 105. Four ESTs were found in support of this variant (asterisks). (E) Splicing of E14. The EST database was interrogated with the sequences corresponding to E13–E15. The boundary between E13 and E15 is placed at bp 168. One EST was found in which E14 was spliced out (asterisk). (F) Splicing of E13+E14. The EST database was interrogated with the sequences corresponding to E2–E15. The boundary between E12 and E15 is placed at bp 105. Four ESTs was found in which E13 and E14 were spliced out together (asterisks). (G) Splicing of E22. The EST database was interrogated with the sequences corresponding to E21–E23–E25. The boundary between E21 and E23 is placed at bp 136. Note the absence of EST clones splicing out E22. The clone marked with a black arrow head is anomalous, it contains E21 in sense fused to a fragment composed of E23+E24+E25 plus part of the coding region of E26 in antisense. The clones marked with red arrow heads splice in the 31 bp variant of E24 (tick mark at bp 184). (H) Splicing of E24. The EST database was interrogated with the sequences corresponding to E23–E24–E25 using the 31 bp variant of E24 (nucleotides 49–79 in the graph). Inspection of the six top hit alignments revealed three that include the 31 bp variant and three that include the 13 bp variant (the 18 bp difference is too short to be displayed in the graph). Five ESTs matching almost only E24 at the bottom of the graph (in green) probably correspond to intron-retaining transcripts (PPP1R12A-210 or 220).

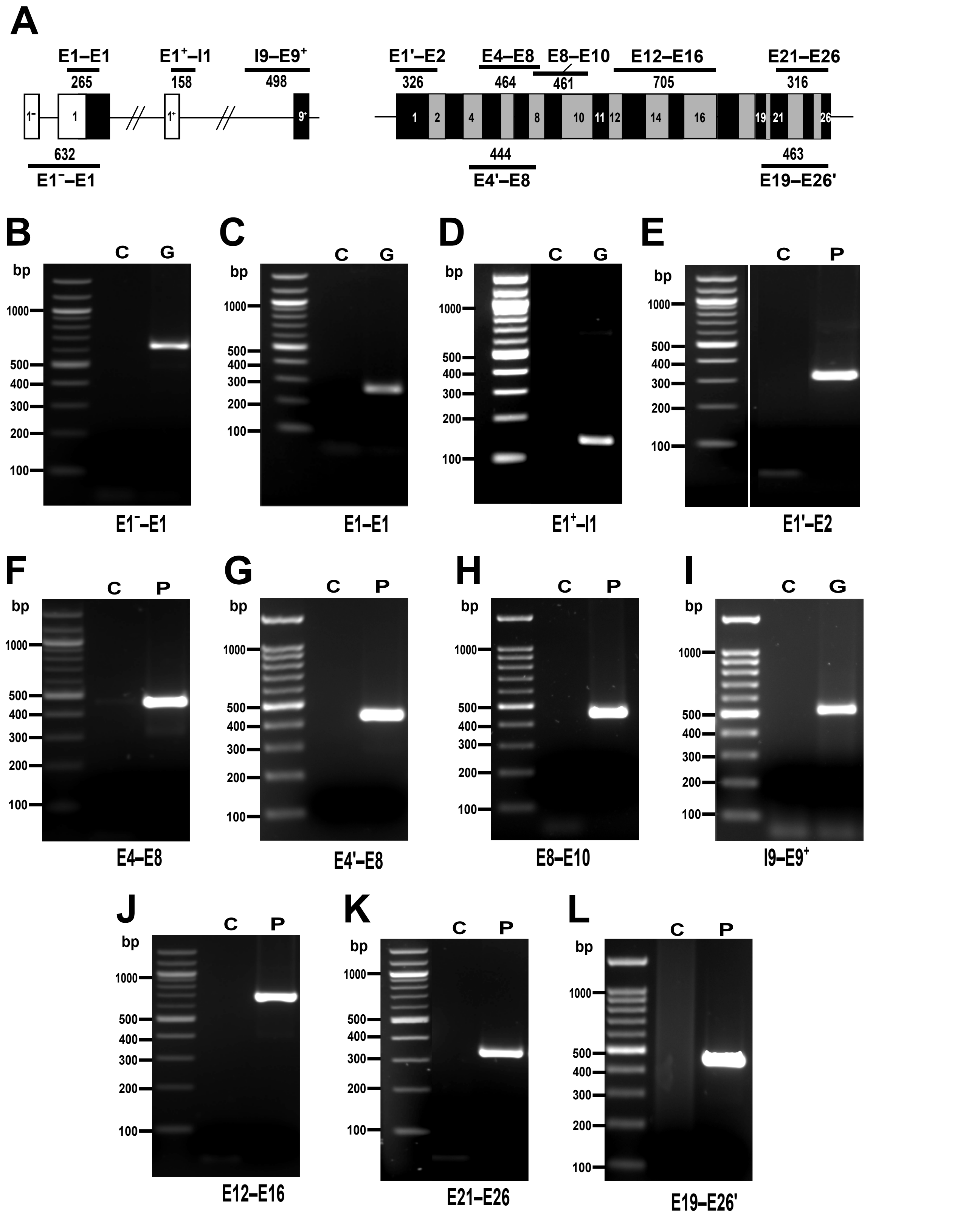

Figure S5. Test PCR reactions of *PPP1R12A* primers. (A) Position and expected size of PCR products. On the left, scheme of the genomic sequence around exons E1 and $E9^{+}$; white and black boxes represent untranslated and translated regions, respectively. On the right, coding region of the canonical *PPP1R12A* FL variant (lacking E24); exons are shown at scale alternating black and grey. Primer pairs in the forward-reverse sequence and size of expected PCR products are indicated above and under the DNA schemes. Primer sequences and PCR conditions are shown in Table S1. (B-L) Results of PCR reactions using the primer combinations of the top panel. G, reactions were run using genomic DNA; P, reactions were run using a plasmid carrying the coding sequence of the FL *PPP1R12A* variant as template; C, control reaction without template.

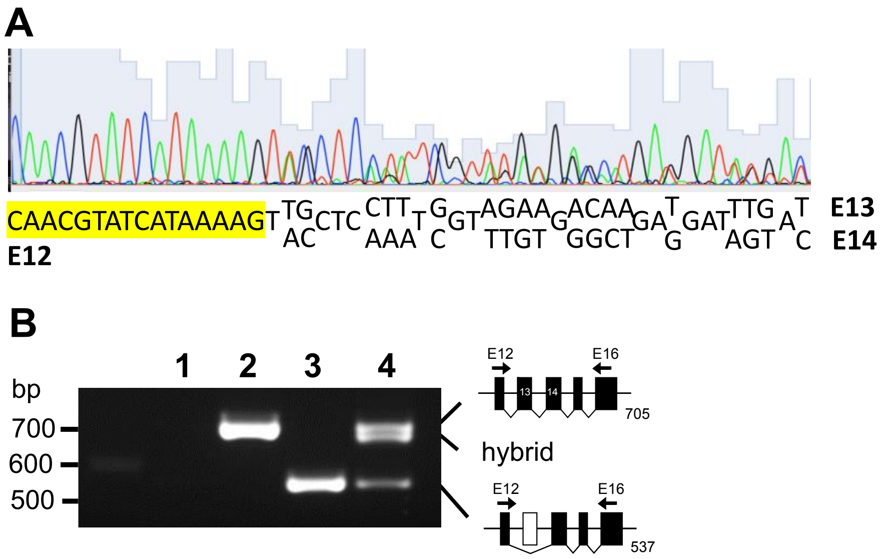

Figure S6. Hybridization of PCR products of reaction targeting E13 and E14 variants. (A) Fragment of the sequencing reaction chromatogram of a hybrid product (intermediate band) of the PCR reaction with primer pair E12-E16 (Fig. 5D). The hybrid band was purified from an agarose gel and sequenced with E12 forward primer. The 3’ end of E12 is highlighted in yellow. It is followed by mixed sequences compatible with E13 and E14, confirming that the band is a hybrid of E13in and E13out DNA fragments. (B) Hybridization of E13in and E13out amplicons in a PCR reaction using plasmid DNA as template. Lane 1, negative control (no template); lane 2, plasmid containing full length MYPT1 cDNA as template; lane 3, plasmid containing an MYPT1 cDNA fragment lacking E13 as template; lane 4, mix of both plasmids as template.

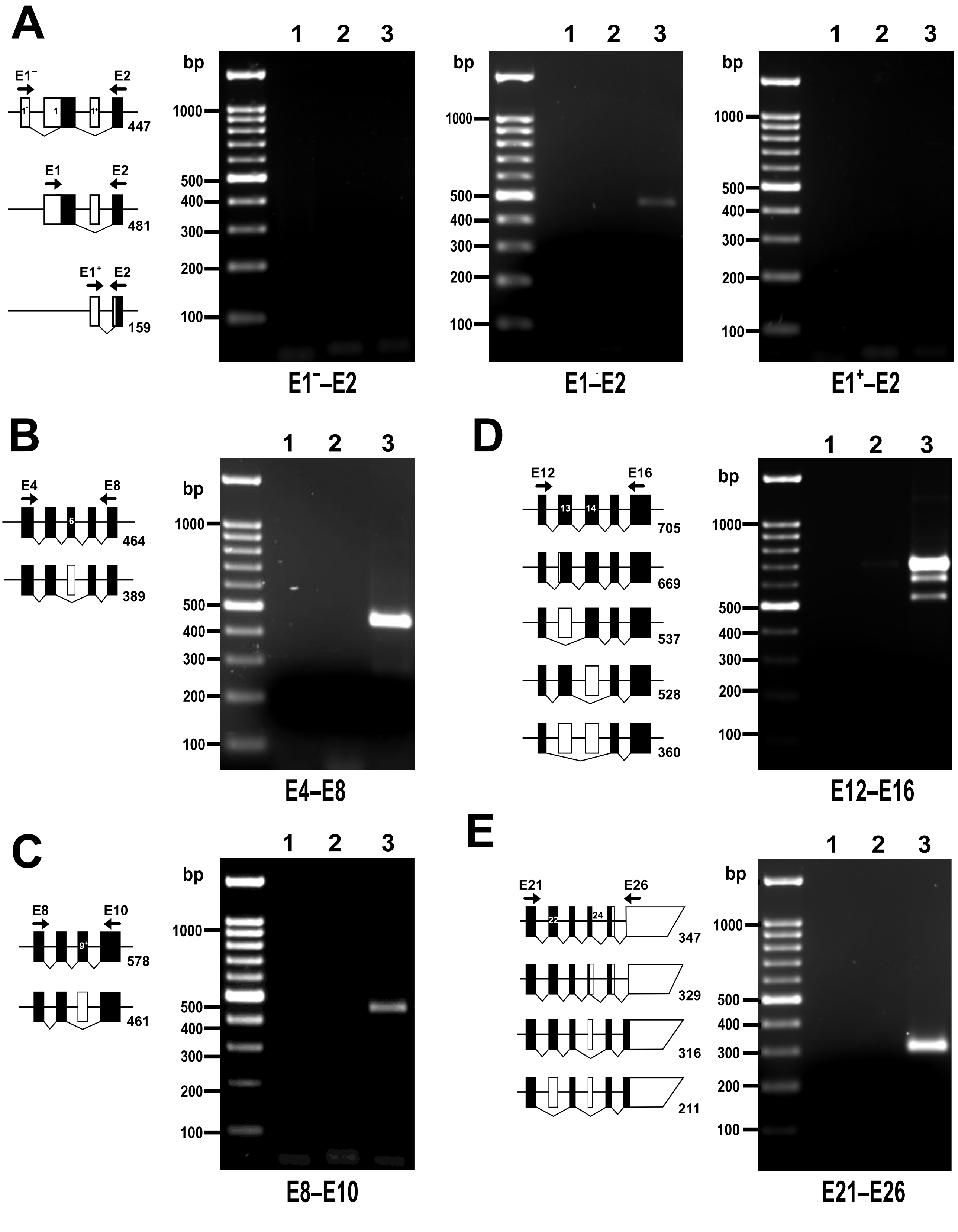

Figure S7. *PPP1R12A* transcripts in human saphenous vein smooth muscle cells as determined by RT-PCR. The diagrams accompanying each panel depict the expected size of PCR products corresponding to all possible alternatively spliced variants sorted by size. White and black boxes represent untranslated and translated exons, respectively. Exons are depicted at scale. Position of primer pairs is indicated schematically. (A) PCR reactions targeting alternative transcription start sites. A transcription start on E1 is the only site used by HSVSMCs. PCR reactions targeting (B) E6, (C) $E9^{+}$, (D) E13 and E14, (E) E22 and E24 splicing variants. PCR reactions were run using no template (lane 1), negative control for reverse transcriptase (lane 2) or cDNA (lane 3) as templates. Primer sequences and PCR conditions are shown in Table S2.

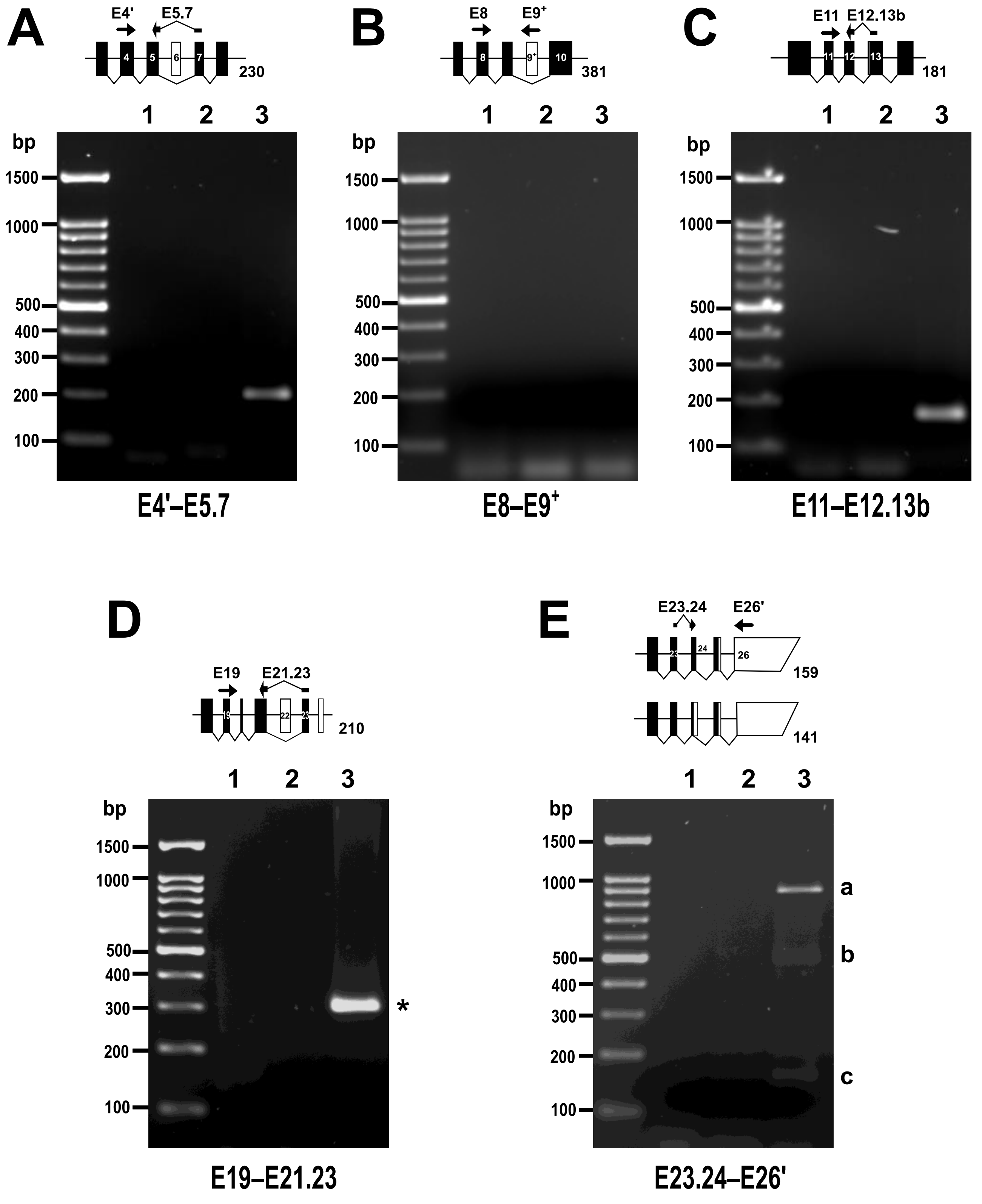

Figure S8. Rare *PPP1R12A* transcripts in human saphenous vein smooth muscle cells as determined by RT-PCR. The diagrams accompanying each panel depict the expected size of PCR products corresponding to the rare alternatively spliced variants. White and black boxes represent untranslated and translated exons, respectively. Exons are depicted at scale. Position of primer pairs is indicated schematically. PCR reactions targeting the (A) ΔE6, (B) E9$^+$, (C) E13b and (D) ΔE22 splicing variants. The asterisk indicates an unexpected PCR product. (E) PCR reaction targeting LZ$^-$ splicing variants. Bands a and b are unexpected amplicons, band c corresponds to the 159 bp product. PCR reactions were run using no template (lane 1), negative control for reverse transcriptase (lane 2) or cDNA (lane 3) as templates. Primer sequences and PCR conditions are shown in Table S2.

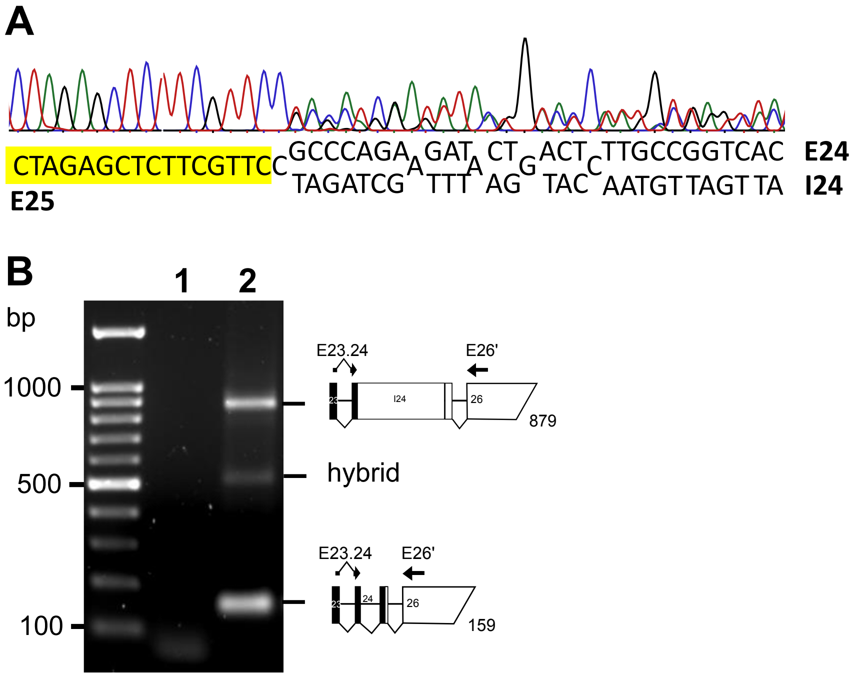

Figure S9. Hybridization of PCR products of the reaction targeting E24. (A) Fragment of the sequencing reaction chromatogram of a hybrid product (intermediate band) of the PCR reaction with primer pair E23.24–E26' (Fig. S8E). The hybrid band was purified from an agarose gel and sequenced with 26' reverse primer. The 5' end of E25 is highlighted in yellow. It is followed by mixed sequences compatible with E24 and I24, confirming that the band is a hybrid of E24in and retained intron DNA fragments. (B) Hybridization of E24in (159 bp) and a retained intron (879 bp) DNA in a PCR reaction using the hybrid band as template (lane 2). Lane 1 is a negative control (no template). This reaction yielded the 159 bp and the 879 bp along with a small amount of hybrid product.

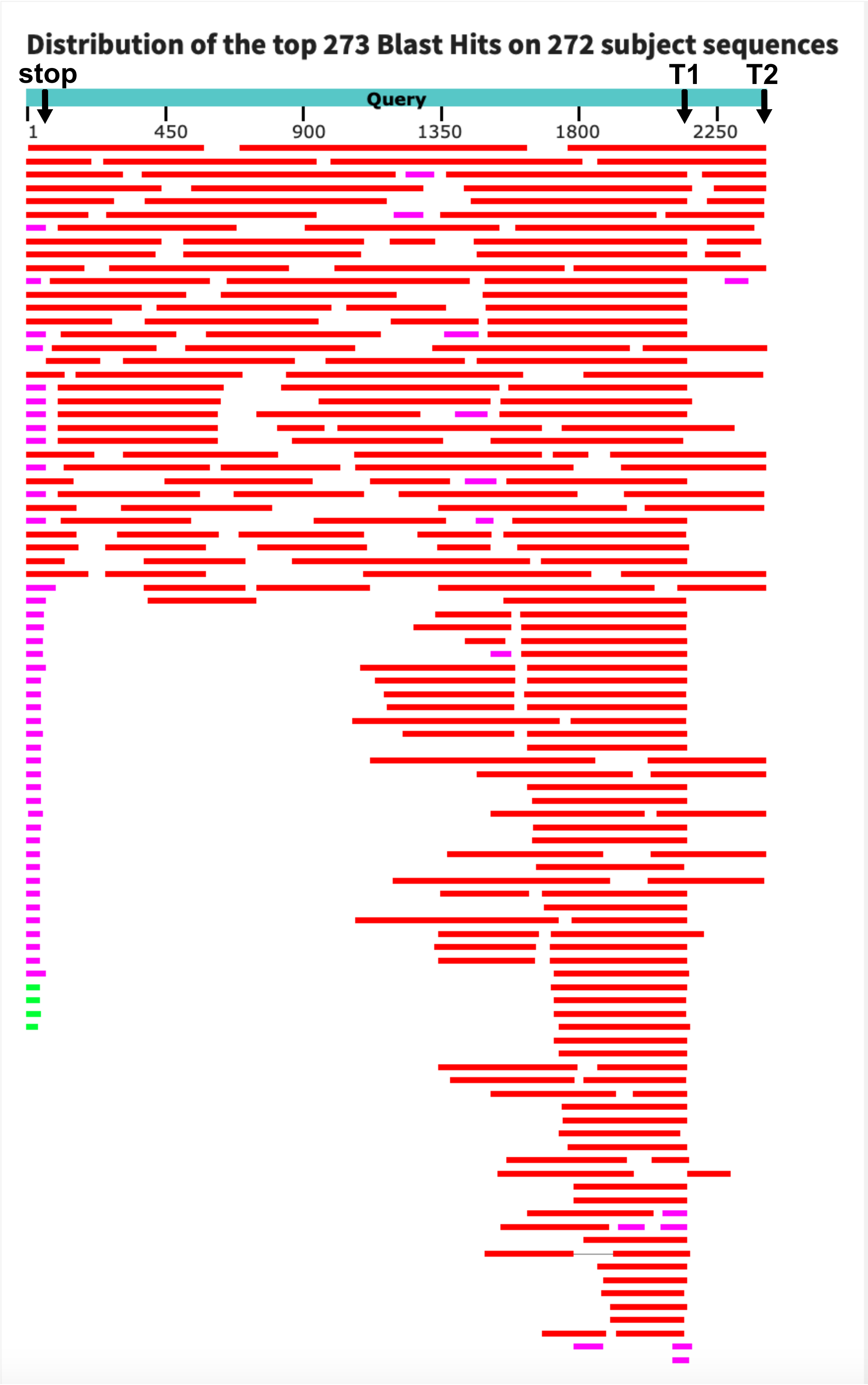

Figure S10. Results of BLASTn search for ESTs in the 3'-UTR region of *PPP1R12A*. The human EST database was interrogated with a BLASTn search using the sequences corresponding to the last 52 coding bp followed by the complete 3'-UTR. Horizontal bars represent the extent of the alignment of the database sequences to the query sequence and are color-coded by score: red, pink and green from highest to lowest. An accumulation of ESTs that terminate sharply approximately 2100 bp downstream of the stop codon suggests the presence of a terminator in that area (T1). A less common terminator (T2) is suggested 260 bp downstream of the first termination site.
